# New freshwater mussels from two Southeast Asian genera *Bineurus* and *Thaiconcha* (Pseudodontini, Gonideinae, Unionidae)

**DOI:** 10.1038/s41598-021-87633-w

**Published:** 2021-05-10

**Authors:** Ekaterina S. Konopleva, Ivan N. Bolotov, John M. Pfeiffer, Ilya V. Vikhrev, Alexander V. Kondakov, Mikhail Yu. Gofarov, Alena A. Tomilova, Kitti Tanmuangpak, Sakboworn Tumpeesuwan

**Affiliations:** 1 Northern Arctic Federal University, Arkhangelsk, Russia; 2 N. Laverov Federal Center for Integrated Arctic Research of the Ural Branch of the Russian Academy of Sciences, Arkhangelsk, Russia; 3 National Museum of Natural History, Smithsonian Institution, Washington, DC USA; 4 Department of Science, Faculty of Science and Technology, Loei Rajabhat University, Loei, Thailand; 5 Department of Biology, Faculty of Science, Mahasarakham University, Maha Sarakham, Thailand

**Keywords:** Zoology, Taxonomy

## Abstract

The Mekong and Chao Phraya rivers harbor a species-rich freshwater mussel assemblage containing a large radiation of the Pseudodontini species. Members of the genera *Bineurus* Simpson 1900 and *Thaiconcha* Bolotov et al., 2020 primarily inhabit small and medium-sized tributaries of these rivers. Here, we present an integrative taxonomic review of these genus-level clades. We show that *Bineurus* contains four species: *B. mouhotii* (Lea, 1863), *B. exilis* (Morelet, 1866) stat. rev., *B. anodontinum* (Rochebrune, 1882) stat. rev., and *B. loeiensis* sp. nov. In its turn, *Thaiconcha* comprises three species: *T. callifera* (Martens, 1860), *T. munelliptica* sp. nov., and *T. thaiensis* sp. nov. Two species, *Pseudodon ovalis* Morlet, 1889 and *P. thomsoni* Morlet, 1884, are considered here as questionable taxa. These findings further highlight that Southeast Asia represents a significant evolutionary hotspot of freshwater mussels, which requires further international collaborative research and conservation efforts.

## Introduction

The Mekong is the largest freshwater basin in Southeast Asia harboring a species-rich fauna of the Unionidae^1–3^. Several recent phylogenetic studies have substantially revised the systematics of various freshwater mussel taxa from the Mekong basin, especially among representatives of the tribes Indochinellini (e.g. *Scabies* Haas, 1911^4, 5^, *Harmandia* Rochebrune, 1882^4^, and *Unionetta* Haas, 1955^4^), Rectidentini (*Ensidens* Frierson, 1911^6, 7^, and *Hyriopsis* Conrad, 1853^3, 7, 8^), and Contradentini (*Contradens* Haas, 1911^7, 9–11^). Less taxonomic focus has been paid to the most species-rich tribe in Southeast Asia, the Pseudodontini. It was shown that the genus- and species-level diversity of this clade needs greater systematic attention, as it was largely underestimated^12^.

The genus *Bineurus* Simpson 1900 represents a divergent clade within the Pseudodontini, with at least two valid species: *B. mouhotii* (Lea, 1863) and *B. exilis* (Morelet, 1866)^12^. Originally, it was described as a subgenus within *Pseudodon* Gould, 1844 with three species: *Pseudodon mouhotii*, *P. exilis*, and *P. avae* (Theobald, 1873)^13, 14^. Later, Prashad^15^ transferred *Pseudodon avae* to the genus *Indopseudodon* Prashad, 1922. Afterwards Haas^16^ synonymized *Pseudodon exilis* with *P. mouhotii* and placed only two species in the section *Bineurus,* i.e. *P. mouhotii* and *P. hageni* (Strubell, 1897). *Bineurus* has remained to be a valid subgenus/section until a comprehensive revision of Brandt^1^ who recognized the single genus *Pseudodon* (without subgenera and sections) containing four species, i.e. *P. mouhoti*, *P. inoscularis* (Gould, 1844), *P. cambodjensis* (Petit, 1865), and *P. vondembuschianus* (Lea, 1840) (the last three taxa with several subspecies). Recently, Bolotov et al.^17^ resurrected *Bineurus* as a valid genus based on the results of an integrative taxonomic analysis. However, the species-level taxonomy of *Bineurus* taxa remains poorly understood, and yet to be revised using DNA sequence data. Furthermore, the genus *Thaiconcha* Bolotov et al., 2020 was introduced to separate the *Pseudodon callifera/P. ellipticum* group from the *Bineurus* clade^12^. *Pseudodon callifera* (Martens, 1860) was assigned as the type species for the genus, whereas *P. ellipticum* Conrad, 1865 and *P. thomsoni* Morlet, 1884 were synonymized with the first species^12^. Finally, the nominal taxon *Pseudodon ovalis* Morlet, 1889 was also placed in *Thaiconcha* but that solution was based solely on conchological features^12^.

The present study aims to (1) revise the genera *Bineurus* and *Thaiconcha* using integrative approach combining DNA-based, morphological, and biogeographic evidence, and (2) describe three freshwater mussel species new to science discovered in the Mekong and Chao Phraya basins.

## Results

**Figure 1:**
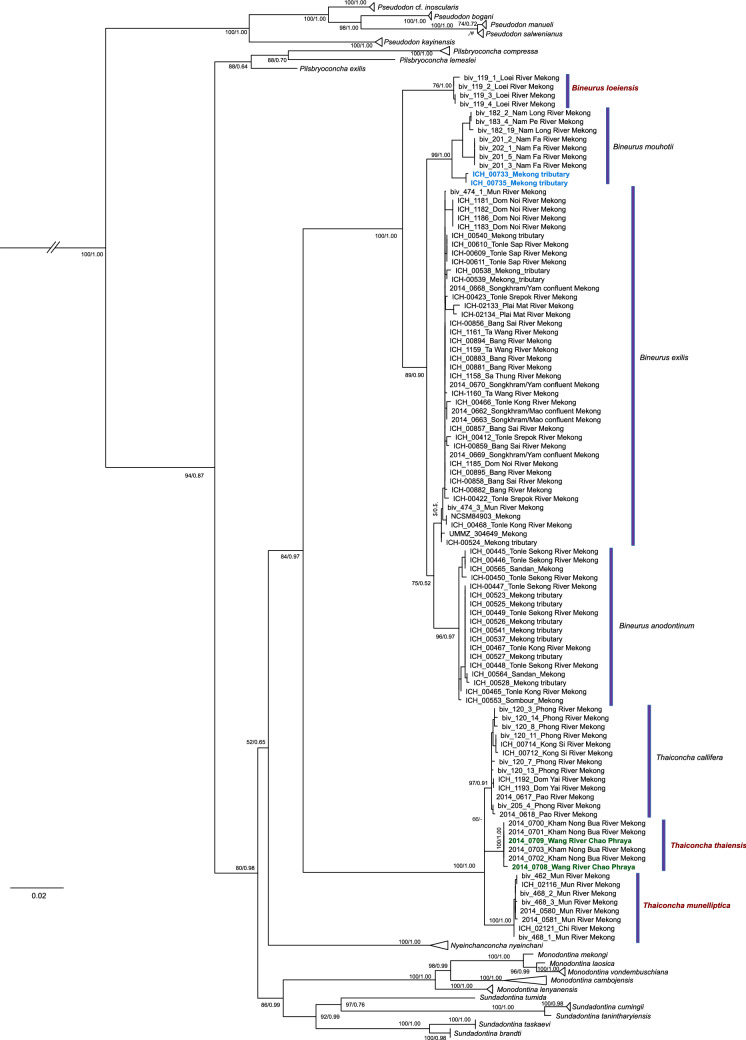
Maximum likelihood phylogeny of the complete data set of mitochondrial and nuclear sequences (five partitions: three codons of *COI* + *16S rRNA* + *28S rRNA*). Outgroup is not shown. Black numbers near nodes are ML Bootstrap Support values (BS)/Bayesian Posterior Probabilities (BPP). The new species names are red; the divergent lineage of *Bineurus mouhotii* is blue; the Chao Phraya’s lineage of *Thaiconcha thaiensis* is green.

Our multi-locus phylogeny based on three molecular markers (*COI* + *16S rRNA* + *28S rRNA*) included 143 individuals from the Pseudodontini (Fig. 1, Supplementary Table 1 and Fig. 1). The tree topology was mainly identical for both the Bayesian and the maximum-likelihood phylogenetic analyses (Fig. 1 and Supplementary Fig. 1). *Bineurus* and *Thaiconcha* were both recovered as strongly supported clades (BS/BPP = 100/1.00). We identified four species-level *Bineurus* subclades corresponding to *B. mouhotii, B. exilis, B. anodontinum*, and *B. loeiensis* sp. nov. The *Thaiconcha* clade contained three species-level subclades, including *T. callifera* and two undescribed lineages, i.e. *T. munelliptica* sp. nov. and *T. thaiensis* sp. nov. All the novel subclades represented well-supported species-level groups with diagnostic nucleotide substitutions (Table 1). The level of genetic divergence (uncorrected *COI* p-distance) among these species varies from 2.4 to 5.8% (Table 1).

**Table 1:** Molecular diagnoses of new *Bineurus* and *Thaiconcha* species from Mekong and Chao Phraya basins.

Species	Mean *COI p*-distance from the nearest neighbor, %	The nearest neighbor of new species	Fixed nucleotide differences based on the sequence alignment of congeners		
			*COI*	*16S rRNA*	*28S rRNA*
*B. loeiensis* sp. nov	5.8	*B. exillis*	41C, 56 A, 95 A, 122 G, 221 C, 275 C, 303 C, 329 A, 398 C, 413 G, 419 C, 486 T, 557 G, 584 C, 590 C, 608 A, 629 T, 644 G	11 G, 15 T, 234 G, 255 T, 390 G, 432 T	653 G
*T. munelliptica* sp. nov	3.1	*T. callifera*	20 G, 41 C, 161 A, 197 A, 293 A, 401 C, 476 G, 558 C, 602 C, 641 C	132 G, 258 G, 259 G, 318 G	n/a
*T. thaiensis* sp. nov	2.4	*T. callifera*	116 C, 134 G, 158 C, 206 C, 264 C, 374 T, 407 C, 617 T	n/a	n/a

**Figure 2:**
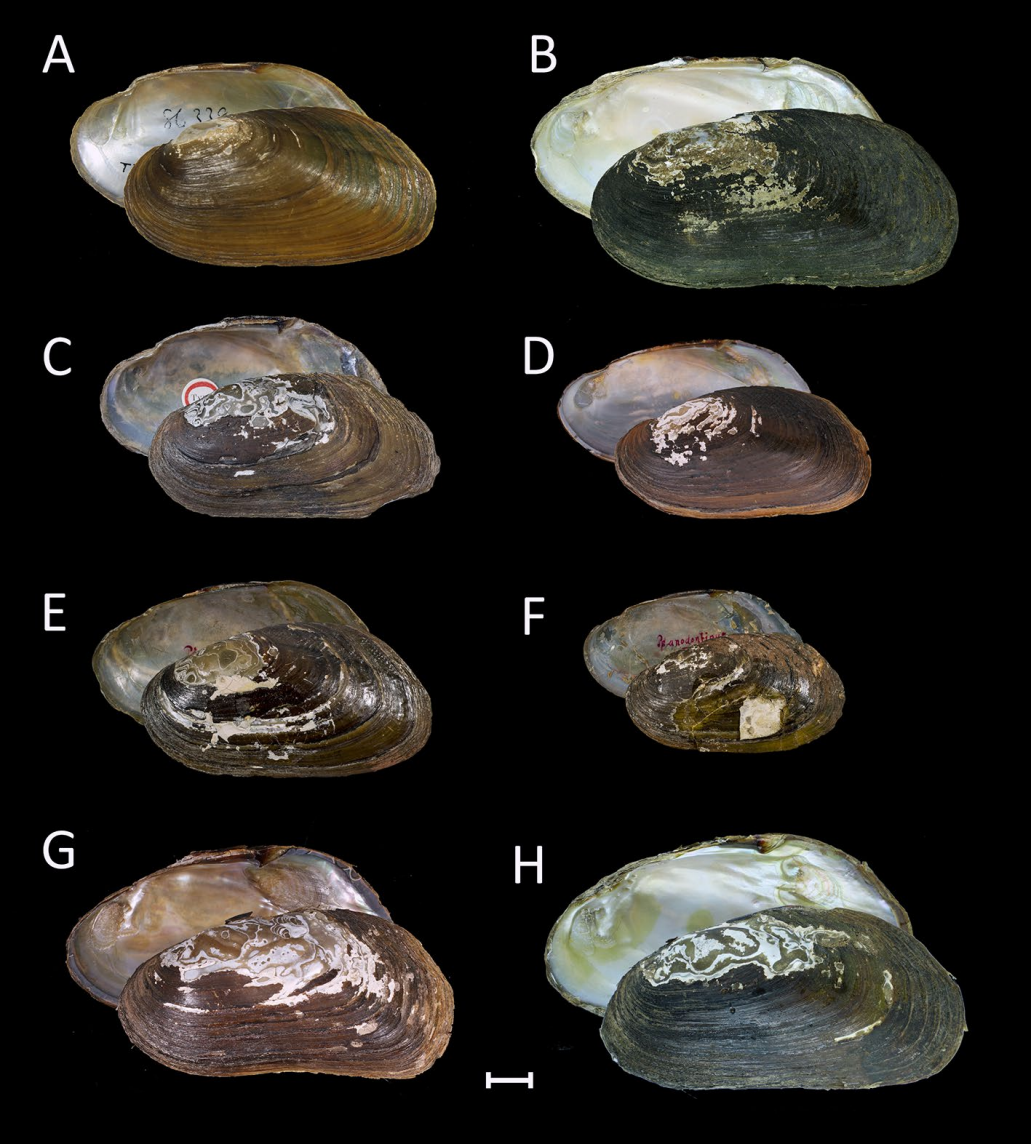
Shells of *Bineurus* species from the Mekong basin. (**A**) *B. mouhotii* (Lea, 1863) [holotype USNM 86339]; (**B**) *B. mouhotii* [RMBH biv182_19, Nam Long River, Nam Ou basin, Mekong, Laos]; (**C**) *B. exilis* (Morelet, 1866) stat. rev. [lectotype NHMUK 1893.2.4.1981]; (**D**) *B. exilis* stat. rev. [topotype UF 507440 (ICH-00610), tributary of Tonle Sap River, Mekong River basin, Cambodia]; (**E**) *Pseudodon pierrei* Rochebrune, 1882 syn. nov. [syntype MNHN-IM-2000-1760] (= *B. exilis*); (**F**) *B. anodontinum* (Rochebrune, 1882) stat. rev. [syntype MNHN-IM-2000-1638]; (**G**) *B. anodontinum* stat. rev. [topotype UF 507419 (ICH-00553), Mekong River basin, Sambour, Cambodia]; (**H**) *B. loeiensis* sp. nov. [holotype RMBH biv119_1, Loei River, Thailand]. Scale bar = 1 cm. Photos: Ilya V. Vikhrev [A]; Ekaterina S. Konopleva [B, H]; Kevin Webb (NHMUK Photographic Unit) [C]; Manuel Caballer (ANR-11-INBS-0004; 2018 MNHN Project “RECOLNAT”) [E, F]; and John M. Pfeiffer [D, G].

Past studies on the genus *Bineurus* have demonstrated that species within this clade are difficult to distinguish morphologically^1, 17^. All four *Bineurus* species are characterized by having rhomboidal or kidney-like shell outlines, rather thin and compressed shells, and tubercle-like pseudocardinal teeth (Fig. 2). Prospective topotypes of *Monocondylaea exilis* Morelet 1866 from a tributary of the Tonle Sap River in Cambodia were generally similar to the lectotype of this nominal taxon, according to the shell shape, hinge plate, and underdeveloped muscle scars, although several topotypes share wrinkles on the posterior margin. The nominal taxon *Pseudodon pierrei* Rochebrune, 1882 synonymized with *Bineurus exilis* also shared the same features. Prospective topotypes of *Pseudodon anodontinum* Rochebrune, 1882 found in the Lower Mekong upstream of Sambour and genetically similar specimens from other localities of this river were rather variable in the shell shape but most of these samples had well-developed posterior muscle attachment scars even in young individuals. The latter feature was also observed in a syntype MNHN-IM-2000-1638 of *Pseudodon anodontinum* Rochebrune, 1882. *Bineurus mouhotii* is somewhat similar to *B. anodontinum* by conchological traits but its geographic distribution is primarily restricted to the Upper Mekong. All generic features are also shared by an undescribed lineage of *Bineurus* (*B. loeiensis* sp. nov.), and it is characterized by having a smooth periostracum without wrinkles, and higher posterior margin.

**Figure 3:**
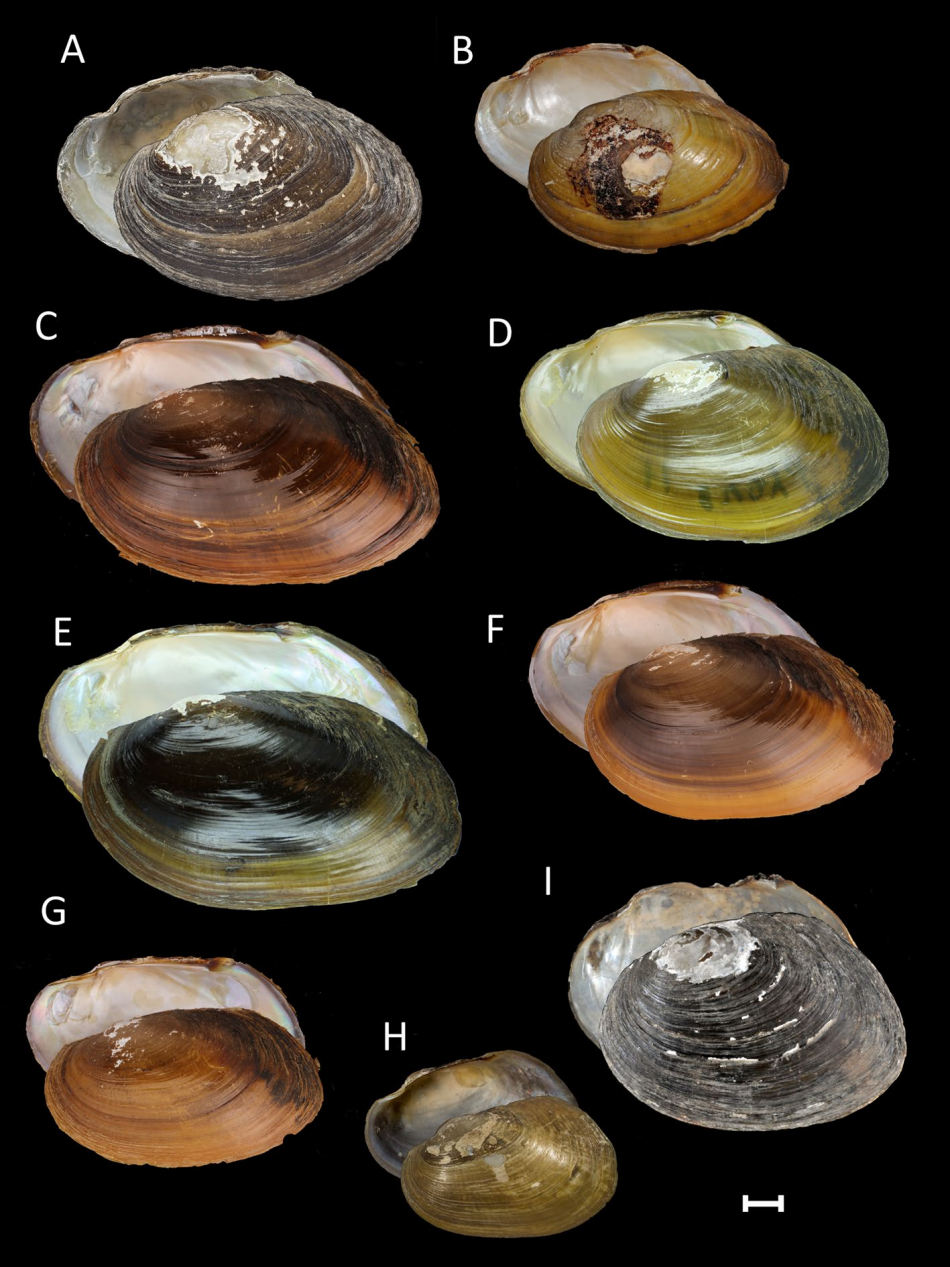
Shells of *Thaiconcha* species from the Mekong basin and questionable taxa. (**A**) *T. callifera* (Martens, 1860) [syntype NHMUK 1859-8-1-20]; (**B**) *Pseudodon ellipticum* Conrad, 1865 [syntype ANSP 41763] (= *T. callifera*); (**C**) *T. callifera* [UF 507741 (ICH-00712), Kong Si River, Mekong River basin, Thailand]; (**D**) *T. callifera* [RMBH biv120_7, Phong River, Mekong River basin, Thailand]; (**E**) *T. munelliptica* sp. nov. [holotype RMBH biv468_2, Mun River upstream of Tha Tum village, Mekong River basin, Thailand]; (**F**) *T. munelliptica* sp. nov. [UF 507607 (2014-0580), Mun River, Thailand]; (**G**) *T. thaiensis* sp. nov. [holotype UF 567706 (2014-0700), Kham Nong Bua River, Mekong River basin, Thailand]. (**H**) *Pseudodon thomsoni* Morlet, 1884 [syntype MNHN-IM-2000-1800]; (**I**) *Pseudodon ovalis* Morlet, 1889 [syntype MNHN-IM-2000-35801]. Scale bar = 1 cm. Photos: Kevin Webb (NHMUK Photographic Unit) [A]; ANSP database [B]; John M. Pfeiffer [C, F, G]; Manuel Caballer (ANR-11-INBS-0004; 2018 MNHN Project “RECOLNAT”) [H]; Philippe Maestrati (MNHN) [I]; and Ekaterina S. Konopleva [D, E].

**Figure 4:**
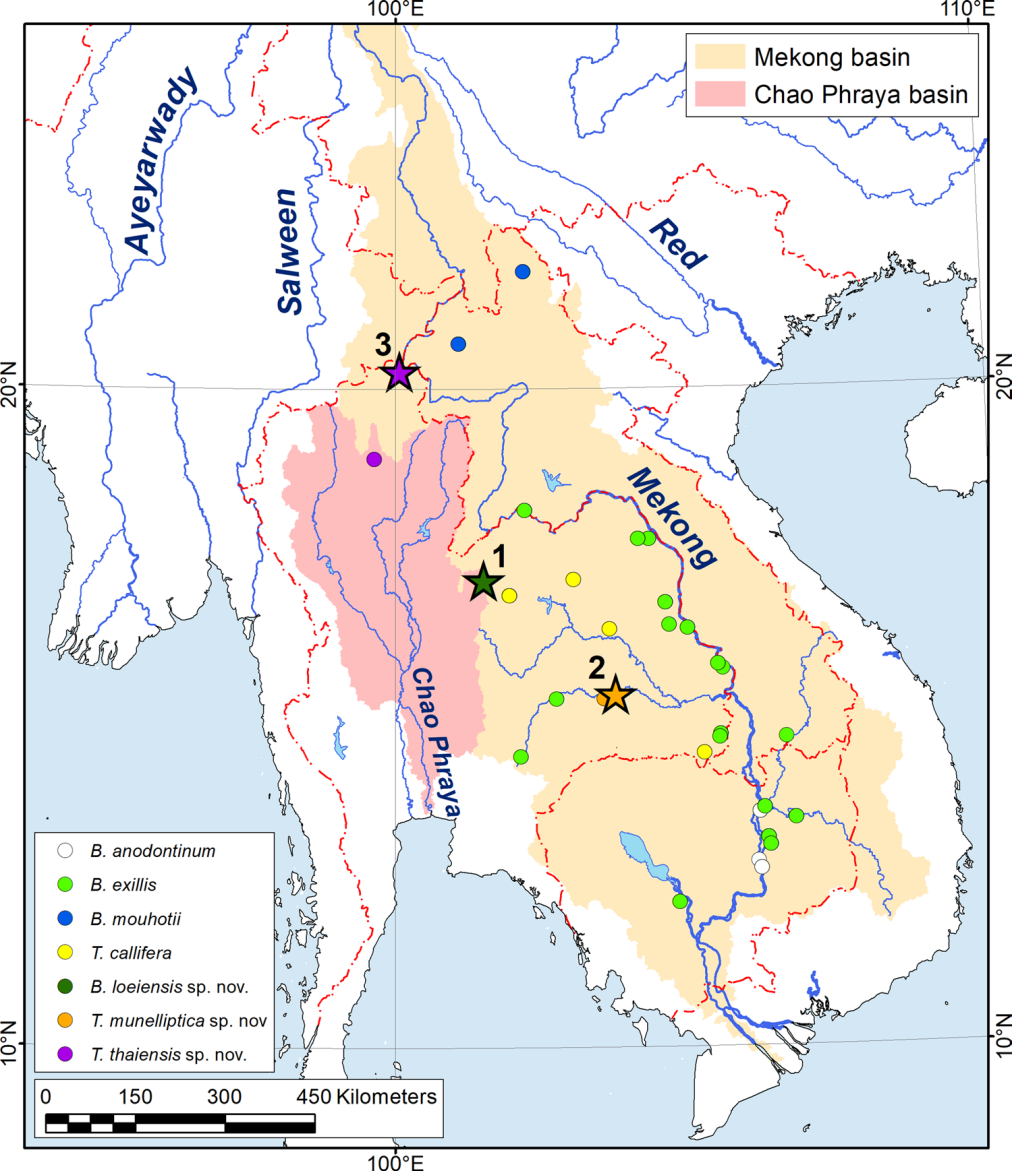
Distribution ranges of *Bineurus* and *Thaiconcha* from Mekong and Chao Phraya basins. The type localities of new species are marked by stars: 1—*Bineurus loeiensis* sp. nov., Loei River, Thailand; 2—*Thaiconcha munelliptica* sp. nov., Mun River, Thailand; 3—*Thaiconcha thaiensis* sp. nov., Kham Nong Bua River, Thailand. The corresponding river basins are highlighted in color. The map was developed using ESRI ArcGIS 10 software (www.esri.com/arcgis). The topographic base of the map was compiled with Natural Earth Free Vector and Raster Map Data (www.naturalearthdata.com), GSHHG version 2.3.7 (http://www.soest.hawaii.edu/pwessel/gshhg)^18^, and the HydroSHEDS database (http://www.hydrosheds.org)^19, 20^. (Map: Mikhail Yu. Gofarov).

The *Thaiconcha* species share elliptical shells, small umbo, more or less developed pseudocardinal teeth and muscle scars, and elongated posterior end (Fig. 3). Among *Thaiconcha callifera* specimens, there were two primary conchological forms. The first form contained individuals with more circular shell and low, triangular posterior wing, thus being similar to the syntype of this nominal taxon (Fig. 3D). The second form occurred more commonly, and mainly had an elongated, elliptical shell (Fig. 3C). An undescribed lineage of *Thaiconcha* from the Mun basin (i.e. *T. munelliptica* sp. nov.) (Figs. 3E,F, 4) differs from other taxa by having a larger size and greater inflation. Young specimens usually have winged posterior margin but this feature is typically lost with age. The shell of *Thaiconcha thaiensis* sp. nov. is broadly rounded in the posterior margin, and has a less prominent posterior wing.

**Figure 5:**
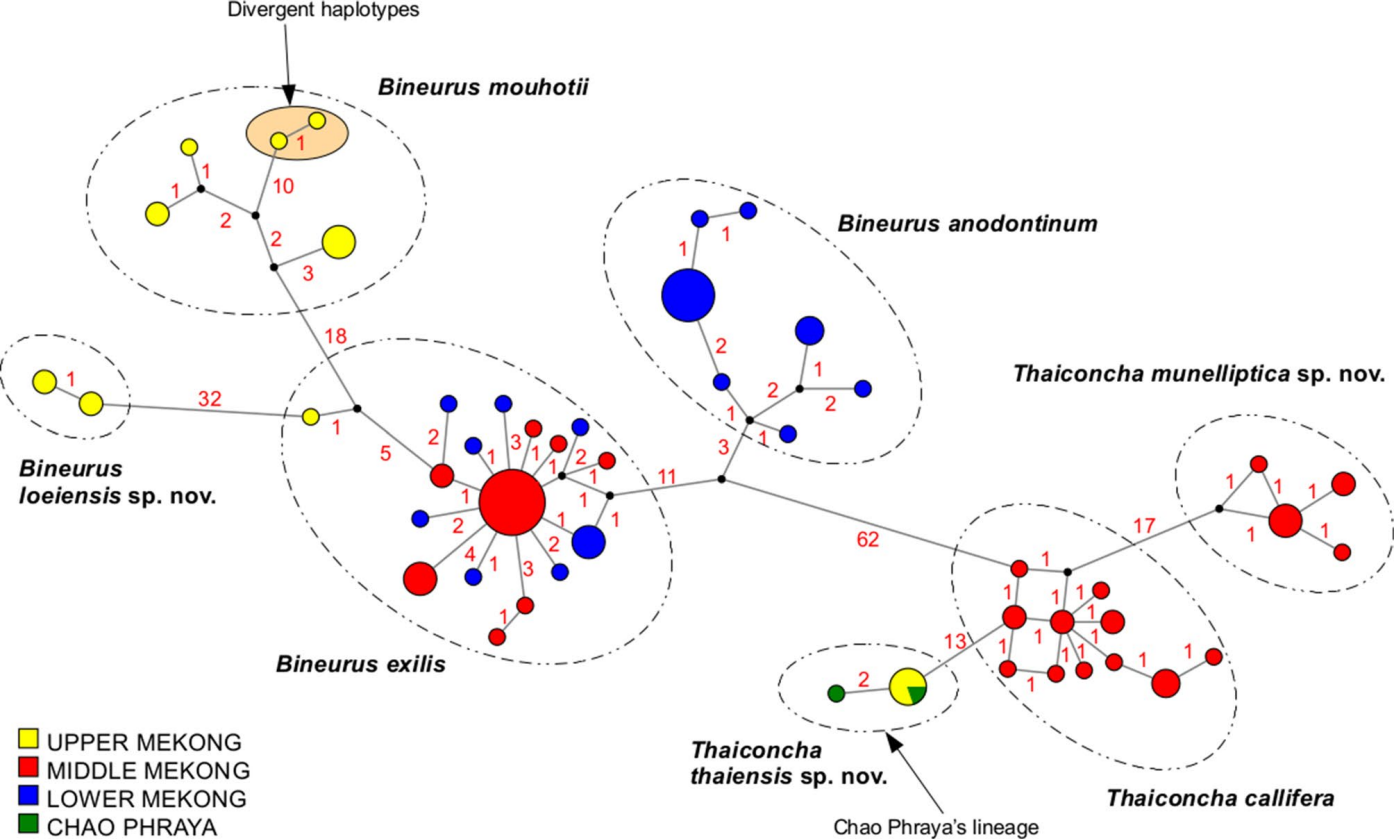
Median joining network of the *COI* sequences of *Bineurus* and *Thaiconcha* species (*N* = 102). The red numbers near branches indicate the numbers of nucleotide substitutions between haplotypes. Size of circles corresponds to the number of available sequences for each haplotype (smallest circle = one sequence). The Mekong was divided into sections based on Halls and Kshatriya^21, 22^.

The range of *Bineurus* includes the Mekong basin in Laos, Thailand, Cambodia, and southern Vietnam. *Bineurus exilis* is the most widespread species, and is distributed throughout the Middle and Lower Mekong (Fig. 4). *Bineurus anodontinum* inhabits small tributaries of the Lower Mekong in Cambodia. *Bineurus mouhotii* is distributed primarily in the Upper Mekong basin in Laos. However, there is a *Bineurus mouhotii* population (catalog No. UF 507756) around Nong Khai in Thailand (18.1511° N, 102.1790° E), although it represents a rather divergent intraspecific lineage (Fig. 5). An undescribed *Bineurus* lineage (*B. loeiensis* sp. nov.) was discovered from the Loei River, a tributary of the Upper Mekong (Fig. 4). We assume that this new species represents an endemic lineage to this watershed.

The *Thaiconcha* taxa mainly inhabit the Middle Mekong basin, although one species (*T. thaiensis* sp. nov.) also occurs in at least one northern tributary of the Chao Phraya (Fig. 4). The *T. thaiensis* sp. nov. specimens (catalog No. UF 507663) from the Chao Phraya population were genetically identical, or very similar (2 nucleotide difference), to specimens from the Kong Si River of the Upper Mekong (Fig. 5).

Additional morphological research using the type specimens of *Pseudodon thomsoni* Morlet, 1884 (syntype Cat. no. MNHN-IM-2000-1800) and *P. ovalis* Morlet, 1889 (syntype Cat. no. MNHN-IM-2000-35801) have demonstrated that these species more likely do not belong to the genus *Thaiconcha*. Both species share a rather specific shell shape that seems to be higher posteriorly than that of *Thaiconcha* taxa. The type specimen of *Pseudodon ovalis* differs from *Thaiconcha* by having a compressed circular shell with weakly developed wing. A syntype of *Pseudodon thomsoni* is obovate, moderately inflated, truncated posteriorly, and has a distinct ridge on the posterior slope. These nominal taxa seem to be representatives of *Monodontina* Conrad, 1853. However, due to the lack of molecular data we transfer these taxa to the questionable group (see Taxonomic account below).

Finally, a taxonomic key was designed for more accurate identification of the genera, belonging to the tribe Pseudodontini.

### Key to the Pseudodontini genera based on conchological features

**Table:** 

1	Shell rhomboidal, compressed, very thin, no teeth…	*Pilsbryoconcha*
–	Shell of other shape, pseudocardinal teeth present…	2
2	Shell elongate-rhomboid or kidney-shaped, usually with concave ventral margin, compressed, with a knob-like pseudocardinal tooth in each valve…	*Bineurus*
–	Shell elliptical, ovate or rounded, ventral margin not concave (convex or straight)…	3
3	Shell elliptical, elongated posteriorly…	*Thaiconcha*
–	Shell ovate or rounded…	4
4	Pseudocardinal teeth weakly developed, shell winged and truncated posteriorly…	*Monodontina*
–	Pseudocardinal teeth strongly developed, knob-like…	5
5	Shell without prominent wing, rather thick, usually with dark brown or black periostracum…	*Sundadontina*
–	Shell winged or somewhat winged…	6
6	Shell somewhat winged, dorsal and posterior margins usually covered by fine wrinkles…	*Pseudodon*
–	Shell winged, rather thin, usually with light brown periostracum with green radial lines posteriorly; posterior area with weakly developed corrugate plication…	*Nyeinchanconcha*

### Taxonomic account

Family Unionidae Rafinesque, 1820.

Subfamily Gonideinae Ortmann, 1916.

Tribe Pseudodontini Frierson, 1927.

Type genus: *Pseudodon* Gould, 1844 (by original designation).

### Genus *Bineurus* Simpson, 1900

Type species: *Monocondyloea mouhotii* Lea, 1863 (by original designation).

Diagnosis: *Bineurus* can be distinguished from its sister genera by an elongated rhomboid/kidney-shaped, inequilateral, compressed, and rather thin shell, usually with straight or concave ventral margin. Hinge plate narrow, with a small, tubercle-like pseudocardinal tooth in each valve. Muscle scars are usually more or less developed.

Distribution: Mekong River basin.

Comments: This genus contains four species (Table 2), one of which is new to science and described here.

**Table 2:** Taxonomic review of freshwater mussel taxa in the genera *Bineurus* and *Thaiconcha* from Mekong and Chao Phraya basins.

Genus	Species	Type and type locality	Distribution
*Bineurus* Simpson, 1900	*B. mouhotii* (Lea, 1863) [= *Monocondyloea mouhotii* Lea, 1863]	Holotype USNM 86339: Laos Mts., Cambodia, Siam [mountain river/stream belonging to the Mekong River basin between Kenethao (17.6908° N, 101.3775° E) and Luang Prabang (19.8949° N, 102.1334° E), Laos]	Mekong basin in Northern Laos (Nam Ou) and Northern Thailand
	*B. anodontinum* (Rochebrune, 1882) [= *Pseudodon anodontinum* Rochebrune, 1882]	Syntype MNHN-IM-2000-1638: Sombor-Sombor, Mekong, Cochinchine [Mekong River at Sambour, approx. 12.7726° N, 105.9629° E, Cambodia]	Lower Mekong in Cambodia
	*B. exilis* (Morelet, 1866) [= *Monocondylaea exilis* Morelet, 1866; ^a^*Pseudodon pierrei* Rochebrune, 1882]	Lectotype NHMUK 1893.2.4.1981: in torrentibus montanis Cambodia (Label data: Lac Tonli-Sap, Cambodia) [Lake Tonle Sap, Cambodia]	Mekong basin in Thailand, Cambodia and southern Vietnam
	*Bineurus loeiensis* sp. nov	Holotype RMBH biv 119_1: Loei River, 17.0982° N, 101.4814° E, Mekong Basin, Thailand	Mekong basin, Thailand
*Thaiconcha* Bolotov et al., 2020	*T. callifera* (Martens, 1860) [= *Anodonta callifera* Martens, 1860; *Pseudodon ellipticum* Conrad, 1865]	Syntype NHMUK 1859-8-1-20: Siam [Thailand]	Mekong basin in Cambodia and Thailand
	*T. munelliptica* sp. nov	Holotype RMBH biv 468_2: Mun River, upstream of Tha Tum village, 15.3575° N, 103.6637° E, Thailand	Mun and Chi Rivers in Thailand
	*T. thaiensis* sp. nov	Holotype UF 567706 (2014-0700), Kham Nong Bua River, trib. of Mekong River, at Rt. 1016 bridge 0.3 mi west of Rt. 1290, 20.2681° N, 100.0721° E, Chiang Rai, Thailand	Mekong and Chao Phraya basins in Thailand

^a^These nominal taxa were placed to the corresponding genera or to synonymy on the basis of conchological features alone, and they are in need of future molecular study.

### *Bineurus mouhotii* (Lea, 1863)

 = *Monocondyloea mouhotii* Lea (1863): 190^23^.

 = *Monocondylaea mouhotiana* Lea (1866): 65^24^.

 = *Pseudodon* (*Bineurus*) *mouhoti* (Lea, 1863): 190^25^.

 = *Pseudodon mouhoti* (Lea, 1863): 265–266^1^.

Figure 2A,B.

Type and type locality: Holotype USNM 86339 (examined by us; Fig. 2A); Laos Mts., Cambodia, Siam.

Material examined: LAOS: tributary of Nam Fa River near Vieng Phou Kha, 20.6820° N, 101.0794° E, Mekong River basin, 24.v.2016, 6 specimens [RMBH biv 201 and RMBH biv 202, including RMBH biv 201_2, RMBH biv 201_3, RMBH biv 201_5, and RMBH biv 202_1 sequenced], Spitsyn leg.; Nam Long River, 21.7700° N, 102.1863° E, Nam Ou River basin, Mekong drainage, 4–12.v.2012, 2 specimens [RMBH biv 182, including specimens RMBH biv 182_2, biv 182_19 sequenced], Bolotov and Vikhrev leg. (Fig. 2B); Nam Pe River, 21.5905° N, 102.0829° E, Nam Ou basin, Mekong drainage, 4–12.v.2012, 1 specimen [RMBH biv 183_4, sequenced], Bolotov and Vikhrev leg. THAILAND: tributary of Mekong River at Rt. 211 bridge approximately 3 km south of Ban Muang, 18.1511° N, 102.1790° E, Mekong River basin, Nong Khai, 24.i.2016, UF 507756, 3 specimens [ICH-00735, ICH-00733 sequenced], Pfeiffer and Page leg.

Distribution: Mekong River basin in western and northern Laos (including Nam Ou River) and northern Thailand.

Comments: Isaac Lea briefly described two new freshwater mussel species using a shell sample labelled as “Laos Mountains, Cambodia, Siam. Monsieur Mouhot, per H. Cuming, Esq.”, i.e. *Monocondyloea mouhotii* and *Unio laosensis* Lea, 1863 (= *Gibbosula laosensis*; Margaritiferidae)^23^. An expanded re-description of these taxa was published three years later^23^. Henri Mouhot, an adventurous French naturalist, collected this sample somewhere during his travel throughout Siam, Cambodia and Laos in the 1858–1861. Lea^24^ noted that Mouhot’s collection contained only five or six freshwater mussel species with the two new taxa. The collector died near Luang Prabang (Laos) in 1861, two years before the description of the taxa. Mouhot’s field diary and correspondence were published in a two-volume work entitled “Travels in the central parts of Indo-China (Siam), Cambodia, and Laos, during the years 1858, 1859, and 1860”^26, 27^. It is likely that the vague type locality of the two taxa was taken by Lea (1863)^17^ from the general route of Mouhot’s travel. In the field notes, Mouhot^26, 27^ did not mention any samples of freshwater shells, although several samples of land snails and seashells were noted. However, Samuel Stevens, a well-known British naturalist, in a letter to Mouhot’s brother (p. 294)^27^ wrote that Mouhot collected freshwater shells only from the Laos Mountains.

### *Bineurus exilis* (Morelet, 1866) stat. rev

 = *Monocondylaea exilis* Morelet (1866): 63^28^ [lectotype NHMUK 1893.2.4.1981; in torrentibus montanis Cambodia (Label: lac Tonli-Sap, Cambodia)] [CAMBODIA: Lake Tonle Sap; examined by us; Fig. 2C].

 = *Pseudodon mouhoti* (Lea, 1863): 265–266^1^.

 = *Pseudodon pierrei* Rochebrune (1882) syn. nov.: 41^29^ [syntype MNHN-IM-2000-1760: Shigloni Breithon, Cochinchine; examined by us; Fig. 2E].

Figure 2C–E.

Topotypes examined: CAMBODIA: a tributary of Tonle Sap River, 12.1889° N, 104.6610° E, Mekong River basin, 2-7.i.2016, UF 507440, 24 specimens [ICH-00609, ICH-00610, and ICH-00611 sequenced], Pfeiffer and Page leg (Fig. 2D).

Other material examined: CAMBODIA: Tonle Srepok River, 13.4434° N, 106.6033° E, Mekong River basin, Khoum Kbal Romeas, 2-7.i.2016, UF 507381, 3 specimens [ICH-00412, ICH-00422, ICH-00423 sequenced], Pfeiffer and Page leg.; Tonle Kong River, 13.6094° N, 106.0924° E, Krong Stung Treng, 2-7.i.2016, UF 567737, 2 specimens [ICH-00466, ICH-00468 sequenced], Pfeiffer and Page leg.; unknown tributary of Mekong just north of Phumi Prêk Preah, 13.1512° N, 106.1452° E, Mekong River basin, Phumi Prêk Preah, 2-7.i.2016, UF 559380, 1 specimen [ICH-00524 sequenced], Pfeiffer and Page leg.; unknown tributary of Mekong just north of Phumi Prêk Preah, 13.04° N, 106.1808°E, Mekong River basin, Phumi Prêk Preah, 2-7.i.2016, UF 507413, 40 specimens [ICH-00538, ICH-00539, ICH-00540 sequenced], Pfeiffer and Page leg. THAILAND: Songkhram River at confluence with Mao River, 17.7020° N, 104.2558° E, Mekong River basin, Nakhon Phanom, 08.i.2015, UF 507639, 2 specimens [2014-0662, 2014-0663 sequenced], Pfeiffer and Page leg.; Songkhram River at confluence with Yam River, 17.7091° N, 104.0767° E, Mekong River basin, Sakon Nakhon, 08.i.2015, UF 507644, 3 specimens [2014-0668, 2014-0669, 2014-0670 sequenced], Pfeiffer and Page leg.; Bang Sai River at Rt. 2292 bridge, 16.7368° N, 104.5195° E, Mekong River basin, Mukdahan, 29.i.2016, UF 507816, 70 specimens [ICH-00856, ICH-00857, ICH-00858, ICH-00859 sequenced], Pfeiffer and Page leg.; Bang River, 16.3464° N, 104.8832° E, Mekong River basin, Mukdahan, 30.i.2016, UF 507828, 18 specimens [ICH-00881, ICH-00882, ICH-00883 sequenced], Pfeiffer and Page leg.; Bang River, 16.3959° N, 104.5715° E, Mekong River basin, Mukdahan, 31.i.2016, UF 507835, 10 specimens [ICH-00894, ICH-00895 sequenced], Pfeiffer and Page leg.; Sa Thung River, 15.7307° N, 105.4511° E, Mekong River basin, Ubon Ratchathani, 01.ii.2016, UF 507845, 3 specimens [ICH-01158 sequenced], Pfeiffer and Page leg.; Ta Wang River, 15.7929° N, 105.3761° E, Mekong River basin, Ubon Ratchathani, 01.ii.2016, UF 507846, 51 specimens [ICH-01159, ICH-01160, ICH-01161 sequenced], Pfeiffer and Page leg.; Dom Noi River, 14.7286° N, 105.3986° E, Mekong River basin, Ubon Ratchathani, 02.ii.2016, UF 507854, 20 specimens [ICH-01181, ICH-01182, ICH-01183, ICH-01185, ICH-01186 sequenced], Pfeiffer and Page leg.; Plai Mat River, 15.2866° N, 102.6853° E, Mekong River basin, Nakhon Ratchasima, 16-28.i.2017, UF 507476, 2 specimens [ICH-02133, ICH-02134 sequenced], Pfeiffer and Page leg.; a tributary of Mekong, 18.1511° N, 102.1790° E, Mekong River basin, Nakhon Ratchasima, 24.i.2016, UF 541633, 1 specimen [ICH-00734 sequenced], Pfeiffer and Page leg.; upstream of upper reservoir, 14.4138° N, 102.0821° E, Mun River, Mekong River basin, 12.iii.2018, 7 specimens [RMBH biv 474, including RMBH biv 474_1 and RMBH biv 474_3 sequenced], Bolotov and Vikhrev leg.

Distribution: Mekong River basin in Thailand, Cambodia, and southern Vietnam.

Comments: The nominal taxon *Pseudodon pierrei* is considered here to be a junior synonym of *Bineurus exilis* based on conchological similarity of the type specimen (Fig. 2E) and geographic proximity of the type locality.

### *Bineurus anodontinum* (Rochebrune, 1882) stat. rev

 = *Pseudodon anodontinum* Rochebrune (1882): 41^29^.

 = *Pseudodon (Bineurus) mouhoti* (Lea, 1863): 265–266^1^.

 = *Pseudodon mouhoti* (Lea, 1863): 265–266^1^.

Figure 2F,G.

Type and type locality: Syntype MNHN-IM-2000-1638 (examined by us; Fig. 2F); Sombor-Sombor, Mekong, Cochinchine [CAMBODIA: Mekong River at Sambour, approx. 12.7726° N, 105.9629° E].

Topotypes examined: CAMBODIA: an anabranch of Mekong River about 1.4 miles upstream from Sambour, 12.7946° N, 105.9726° E (Fig. 2G), 2-7.i.2016, UF 507419, 3 specimens [ICH-00553 sequenced], Pfeiffer and Page leg.

Other material examined: CAMBODIA: Tonle Sekong River, 13.5450° N, 106.0135° E, Mekong River basin, Krong Stung Treng, 2-7.i.2016, UF 507391, 9 specimens [ICH-00445, ICH-00446, ICH-00447, ICH-00448, ICH-00449, ICH-00450 sequenced], Pfeiffer and Page leg.; Tonle Kong River just upstream of island, 13.6094° N, 106.0924° E, Mekong River basin, Krong Stung Treng, 2-7.i.2016, UF 507896, 4 specimens [ICH-00465, ICH-00467 sequenced], Pfeiffer and Page leg.; unknown tributary of Mekong just north of Phumi Prêk Preah, 13.1512° N, 106.1452° E, Mekong River basin, Phumi Prêk Preah, 2-7.i.2016, UF 507408, 28 specimens [ICH-00523, ICH-00525, ICH-00526, ICH-00527, ICH-00528 sequenced], Pfeiffer and Page leg.; downstream of Sandan, 12.6869° N, 106.0170° E, Mekong River, Sandan, 2-7.i.2016, UF 507424, 5 specimens [ICH-00564, ICH-00565 sequenced], Pfeiffer and Page leg.; unknown tributary of Mekong just north of Phumi Prêk Preah, 13.0401° N, 106.1808° E, Mekong River basin, Phumi Prêk Preah, 2-7.i.2016, UF 559262, 2 specimens [ICH-00537, ICH-00541 sequenced], Pfeiffer and Page leg.

Distribution: Lower Mekong in Cambodia.

Comments: Our samples were linked to this nominal taxon based on conchological similarity to the type specimen and DNA sequences of topotypes.

### *Bineurus loeiensis* sp. nov

Figures 2H, 4.

LSID: http://zoobank.org/urn:lsid:zoobank.org:act:095BDBBE-CE3B-4AE7-A011-06A2DD0CEAC8

Type and type locality: Holotype RMBH biv119_1; THAILAND: Loei River, 17.0982° N, 101.4814° E, Mekong basin, 08.iv.2014, Bolotov and Vikhrev leg.

Paratypes: THAILAND: type locality, same collecting date and collectors, 10 specimens [RMBH biv119_2, RMBH biv119_3, RMBH biv119_4, RMBH biv119_5, RMBH biv119_6, RMBH biv119_7, RMBH biv119_8, RMBH biv119_9, RMBH biv119_10, and RMBH biv119_11].

Etymology: The name of this species is derived from the Loei River in Thailand, its type locality.

Diagnosis: The new species is conchologically and genetically similar to *Bineurus exillis* but differs from it by a more developed pseudocardinal tooth and the lack of prominent wrinkles posteriorly. It can also be distinguished from other *Bineurus* taxa by fixed nucleotide substitutions in the *COI*, *16S rRNA*, and *28S rRNA* gene fragments (Table 1).

Description: Specimens medium-sized: length 36.8–77.1 mm; height 19.5–38.7 mm; width 9.3–21.3 mm. Shell kidney-shaped, inequilateral, moderately thick, rather compressed, rounded anteriorly and truncated posteriorly; dorsal margin slightly curved, ventral margin straight or slightly curved. The umbones not elevated; strongly eroded. Periostracum dark-brown, smooth; nacre whitish with yellow flecks. Pseudocardinal teeth tubercular-like on each valve. Anterior muscle attachment scars rather developed, somewhat drop-like; posterior ones less prominent, rounded.

Habitat and ecology: The species was found only in the Loei River, northern Thailand. It inhabits river sections with silt-sandy substrate, rocks, and boulders.

Distribution: Loei River, northeast Thailand.

Comments: Bolotov et al.^17^ listed this species as *Bineurus* aff. *mouhotii* (Lea, 1863) sp.2.

### Genus *Thaiconcha* Bolotov et al., 2020

Type species: *Anodonta callifera* Martens, 1860 (by original designation).

Diagnosis: Shell large, elliptical, moderately thick, and inflated. Pseudocardinal teeth rather well developed, muscle attachment scars deep.

Distribution: Mekong and Chao Phraya basins in Thailand and Cambodia.

Comments: This genus contains three species (Table 2), two of which are new to science and described here.

### *Thaiconcha callifera* (Martens, 1860)

 = *Anodonta callifera* Martens (1860): 15^30^.

 = *Pseudodon ellipticum* Conrad (1865): 352 [syntype ANSP 41763: Cambodia; examined by us; Fig. 3B]^31^.

 = *Pseudodon inoscularis callifer* (Martens, 1860): 267^1^.

 = *Pseudodon vendembuschianus ellipticus* Conrad, 1860: 270^1^.

Figure 3A–D.

Type and type locality: Syntype NHMUK 1859–8-1–20 (examined by us; Fig. 3A); Siam [THAILAND].

Material examined: THAILAND: Phong River, 16.8616° N, 101.9105° E, Mekong River basin, 09.iv.2014, 17 specimens [RMBH biv 120 and biv 205, including RMBH biv 120_3, biv 120_4, biv 120_7, biv 120_8, biv 120_11, biv 120_12, biv 120_13, biv 120_14, biv 120_15, and biv 205_4 sequenced], Bolotov and Vikhrev leg. (Fig. 3D); Kong Si River at bridge ESE of Ban Kumphawapi, 17.0969° N, 102.9885° E, Mekong River basin, Udon Thani, 21.i.2016, UF 507741, 5 specimens [ICH-00712, ICH-00714 sequenced], Pfeiffer and Page leg. (Fig. 3C); Dom Yai River at Rt. 2248 bridge approx. 15 km ESE of Nam Yuen, 14.4460° N, 105.1211° E, Mekong River basin, Ubon Ratchathani, 02.ii.2016, UF 507860, 5 specimens [ICH-01192, ICH-01193 sequenced], Pfeiffer and Page leg.; Pao River, tributary of Chi River at Rt. 214 bridge, 16.3402° N, 103.5758° E, Mun River drainage, Mekong River basin, Kalasin, 06.i.2015, UF 507621, 8 specimens [2014-0617, 2014-0618 sequenced], Pfeiffer and Page leg.

Distribution: Mekong basin in Cambodia and Thailand.

### *Thaiconcha munelliptica* sp. nov

Figures 3E,F, 4.

LSID: http://zoobank.org/urn:lsid:zoobank.org:act:4591D422-38FB-4FED-A080-B6285E0BC4BC

Type and type locality: Holotype RMBH biv468_1; THAILAND: pool site with clay bottom, Mun River upstream of Tha Tum village, 15.3575° N, 103.6637° E, Mekong basin, Surin Province, 7.iii.2018, Bolotov and Vikhrev leg.

Paratypes: THAILAND: type locality, same collecting date and collectors as for the holotype, 5 specimens [sequenced specimens RMBH biv468_2 and RMBH biv468_3; RMBH biv468_4, RMBH biv468_5, and RMBH biv468_6]; Mun River at Tha Tum village, 15.3297° N, 103.6821° E, Mekong basin, Surin Province, 7.iii.2018, 1 specimen [sequenced RMBH biv462], local villager leg.; Mun River, 15.3304° N, 103.6818° E, Mekong basin, Surin Province, 06.i. 2015, UF 507607, 2 specimens [sequenced 2014-0580, 2014-0581], Pfeiffer and Page leg.; Mun River, 15.2927° N, 103.5074° E, 2.8 km north of Ban Taklang in Na Nong Phai, Surin Province, 16-28.i.2017, UF 507466, 2 specimens [sequenced ICH-02116], Pfeiffer and Page leg.; Chi River, 15.2838° N, 103.4695° E, 3.4 km WNW of Ban Taklang in Tha Muang, Mekong basin, Buri Ram Province, 16-28.i.2017, UF 507470, 1 specimen [sequenced ICH-02121], Pfeiffer and Page leg.

Etymology: The name of this species is a combination of words “Mun” (i.e. Mun River, after its type locality) and “elliptica” (previously known nominal taxon in this genus).

Diagnosis: This new taxon differs from its sister species *Thaiconcha callifera* by having a massive, more oblong, and inflated shell, and less prominent pseudocardinal teeth. It can also be distinguished from the sister taxon by fixed nucleotide substitutions in the *COI* and *16S rRNA* gene fragments (Table 1).

Description: Specimens medium or large; shell measurements: length 51.8–98.3 mm, height 30.6–56.9 mm, width 15.6–35.1 mm. Shell elongate-ovate, inequilateral, moderately thick, somewhat inflated; anterior and posterior margins rounded, dorsal and ventral sides curved. Younger shells thinner with developed posterior wing; the periostracum olive-greenish with clear radial striola stretching from the umbones. Older specimens with dark-brown periostracum and lighter ventral margin. The nacre bluish, shining. The umbones slightly elevated, eroded. The pseudocardinal teeth tubercular in each valve. Adductor muscle scars somewhat drop-like, contiguous with pedal retractor scars. The posterior muscle scars shallow.

Habitat and ecology: Slow flowing sections of medium-sized rivers with clay or other soft substrate.

Distribution: Mun and Chi rivers in northeast Thailand.

### *Thaiconcha thaiensis* sp. nov

Figures 3G, 4.

LSID: http://zoobank.org/urn:lsid:zoobank.org:act:6067A20D-E8A1-4D6E-921B-00BFC0CB1B0F

Type and type locality: Holotype UF 567706; THAILAND: Kham Nong Bua River, trib. of Mekong River, at Rt. 1016 bridge 0.3 mi west of Rt. 1290, 20.2681° N, 100.0721° E, Chiang Rai, 13.i.2015, Pfeiffer and Page leg.

Paratypes: THAILAND: type locality, same collecting date and collectors as holotype, UF 507660, 16 specimens [sequenced 2014-0701, 2014-0702, 2014-0703].

Etymology: The name of this species is derived from the country of Thailand, in which its type locality is situated.

Diagnosis: This taxon is conchologically similar to *Thaiconcha califera* and *T. munelliptica* but it may be distinguished from these taxa by its more yellow periostracum (especially in young individuals), more broadly rounded posterior margin, and less prominent posterior wing. It can also be distinguished from the sister taxon by fixed nucleotide substitutions in the *COI* gene fragment (Table 1).

Description: Specimens small to large: length 34.0–93.8 mm; height 20.5–53.9 mm; width 9.1–28.9 mm. Shell elongate-ovate, inequilateral, moderately thick, somewhat compressed; anterior and posterior margins rounded, dorsal and ventral margins curved. Smaller specimens thinner shelled and more lightly colored, and broadly rounded posteriorly. Larger specimens thicker shelled, darker, and more pointed posteriorly. Radial striola occasionally present, faint. Nacre faintly purple to blue, often with brown stains, strongly reflective. Umbones slightly elevated, commonly eroded (even in small individuals). Lateral teeth absent, large ligamental fosset. Pseudocardinal tooth, one in each valve, mostly smooth, some faint ridges dorsally. Adductor muscles faint, deepening with age, contiguous. Pedal elevator scars distinct, numerous, in straight line.

Habitat and ecology: Small to medium-sized rivers with slow to moderate flow; often found in deeper parts of the stream in stable mud and sand.

Distribution: Mekong and Chao Phraya river basins.

### Questionable Taxa

#### ?*Pseudodon thomsoni* Morlet, 1884

 = *Pseudodon thomsoni* Morlet (1884): 401^32^.

 = *Pseudodon* (*Bineurus*) *thomsoni* Graf & Cummings (2007): 311^33^.

 = *Thaiconcha callifera* Bolotov et al. (2020) [partim]: 10, 14^11^.

Type and type locality: Syntype MNHN-IM-2000-1800 (examined by us; Fig. 3H); Cambodia.

Distribution: Mekong basin in Cambodia.

Comments: Haas^15^ omitted this nominal taxon. There is no available molecular data for it.

#### ?*Pseudodon ovalis* Morlet, 1889

 = *Pseudodon ovalis* Morlet (1889): 166, 197^34^.

 = *Pseudodon* (*Monodontina*) *ellipticus* Haas (1969) 134^16^.

 = *Pseudodon vondembuschianus ellipticus* Brandt (1974): 270^1^.

 = *Thaiconcha ovalis* Bolotov et al. (2020): 10^12^.

Type and type locality: Syntype MNHN-IM-2000-35801 (examined by us; Fig. 3I); Riviere de Srakeo (Siam) [THAILAND: Bang Pakong River].

Distribution: Bang Pakong basin in Thailand.

Comments: No DNA sequences are available for this taxon.

## Discussion

### Taxonomic issues

Study of the morphology, phylogeny and distribution of the Pseudodontini from Mekong and Chao Phraya revealed four distant species-level taxa in the genus *Bineurus* and three such taxa in the genus *Thaiconcha*. Species in both genera are difficult to distinguish based on conchological traits but are genetically distinct and often times, species are allopathically distributed, which aids in their identification. However, at least one species, *Bineurus exilis,* does co-occurs with its congeners: *B. mouhotii* in the northern part of its distribution (Nong Khai Province, Thailand) and *B. anodontium* in the southern part of distribution, (Kratie Province, Cambodia). These species are morphologically indistinguishable but are divergent phylogenetically. Despite the morphological similarity, their sympatry and high level of genetic divergence suggest that these lineages represent biological species and are intrinsically reproductively isolated. The presence of morphologically similar but phylogenetically distant species among unionids has been demonstrated for a number of genera, e.g. *Pleurobema*^35^, *Hyriopsis*^36^, *Sinanodonta*^37–40^, *Fusconaia*^41^, and *Etheria*^42^.

The representatives of the genus *Thaiconcha* are rather variable morphologically and mainly differed by shell shape, especially within *T. callifera*. Such shape plasticity could be driven by environmental conditions and hydrology which may influence the conchological characteristics^43–45^. *Bineurus* and *Thaiconcha* species were mainly collected from small and medium sized rivers, as well as at the confluence of small tributaries of the Mekong proper, usually with soft (clay, sandy, or somewhat silty) substrate. Less specimens were found in the mainstreams of the Mekong and Chao Phraya.

Our analyses demonstrated a well-resolved phylogeny with high and moderate supports for all available taxa under discussion. Three species new to science, i.e. *Bineurus loeiensis* sp. nov.*, Thaiconcha munelliptica* sp. nov. and *T. thaiensis* sp. nov. were recognized and described under the framework of this study. The minimum level of genetic divergence was 2.4% (Table 1), what is generally appropriate for the species delimitation^41, 46^. The lineage of *Bineurus mouhotii* from a Mekong’s tributary around Nong Khai (catalog No. UF 507756) is rather divergent phylogenetically (based on the COI gene p-distance = 2.2%), and it may belong to a separate taxon (Fig. 5). However, the morphological similarity and the lack of sequences for *16S rRNA* and *28S rRNA* gene fragments preclude any final solution on the taxonomic status of this lineage (Supplementary Table 1).

Based on conchological analyses of the type specimens, *Pseudodon ovalis* and *P. thomsoni* have been transferred to the group of questionable taxa. Features such as winged and circular shell, small umbo, and weakly developed pseudocardinal teeth are not specific for *Thaiconcha* members. However, the type of *Pseudodon thomsoni* represents a young individual that complicates morphological studies, because small specimens of *Thaiconcha* spp. can often be winged. The taxonomic status of both species is yet to be confirmed based on DNA sequence data.

### Distributional and biogeographic notes

The genera *Bineurus* and *Thaiconcha* are primarily restricted to the Mekong basin but *T. thainesis* sp. nov. has also been found in the Upper Chao Phraya basin. This disjunct population suggests a historical stream capture event between the Mekong and Upper Chao Phraya drainages. The hypothesis of past connections between upper parts of these large river basins was supported by a wide array of papers concerning mussels^7^ and fishes^47, 48^, as well as summarized in several biogeographic works^49, 50^. Genetically related snakehead populations of *Channa striata* (Bloch, 1793) were observed in the Mekong (Chiang Rai) and Chao Phraya (Chiang Mai, Lumphun, Sukhothai and others in the northern and central parts of Thailand)^47^. The cyprinid fish *Garra theunensis* Kottelat, 1998, known from the Mekong basin, was found in the Upper Nan River belonging to the Chao Phraya drainage^48^. Among freshwater mussels, *Lens contradens* (Lea, 1838) represents an opposite example to *Thaiconcha thaiensis* as it primarily ranged throughout the Chao Phraya, with a few geographically disjunctive populations in the Mekong (Ing, Kok, and Loei)^7^.

The rearrangements of Southeast Asian river systems such as separations, connections, and stream captures repeatedly occurred in the past mainly caused by tectonic processes and sea level fluctuations^51–53^. The Wang River represents a part of the Northern Chao Phraya drainage, raising from the Phi Pan Nam Mountain Range and flowing through Chiang Rai and Lumpang to the Tak Province, where it joins the Ping River^54^. The tributaries starting from the northern slopes of this range belong to the Mekong basin, while tributaries of the western slopes fell to the Salween River^55^. The Phi Pan Nam Mountains serve as a geographic barrier separating these large river basins. Hence, a disjunctive range of *Thaiconcha thainesis* sp. nov. may reflect past rearrangements of the northern paleo-Chao Phraya and the paleo-Mekong. A small genetic divergence between the *COI* haplotypes of *Thaiconcha thaiensis* sp. nov. from the Wang River and a tributary of the Upper Mekong indicated that these drainage rearrangements could have been triggered by relatively recent geological events (e.g. Pliocene to Pleistocene)^56^. It was found that *Lens contradens* also shares shallow molecular divergence between its populations from the Mekong and Chao Phraya^7^.

In any case, a more extensive sampling is required for these remote water bodies to reconstruct the history of past drainage rearrangements and to exclude a possibility of recent human-mediated introduction events for given taxa.

## Methods

### Data sampling

The specimens of *Bineurus* and *Thaiconcha* species were collected from different water bodies in Thailand, Laos, and Cambodia (Supplementary Table 1). Among them, prospective topotypes of *Bineurus exilis* from a tributary of the Tonle Sap River in Cambodia and of *B. anodontinum* from the Lower Mekong basin (upstream of Sambour) were collected. We studied the type specimens of nominal taxa under discussion and shell lots of other species in the following museum collections: Natural History Museum, London, Great Britain (BMNH); National Museum of Natural History, Smithsonian Institution, Washington D.C., USA (NMNH); Muséum national d’histoire naturelle, Paris, France (MNHN); Florida Museum of Natural History, Gainesville, USA (UF); and Russian Museum of Biodiversity Hotspots, Federal Center for Integrated Arctic Research of the Ural Branch of the Russian Academy of Sciences, Arkhangelsk, Russia (RMBH). Images of the type specimens were obtained from Academy of Natural Sciences of Drexel University, Philadelphia, USA (ANSP), BMNH, and MNHN.

### DNA sequence data and phylogenetic analyses

Molecular analysis was based on three molecular markers, i.e. the *COI*, *16S rRNA*, and *28S rRNA* gene fragments. It was carried out using PCR primers and laboratory protocols as described in our earlier papers^10, 17, 57^. The newly generated sequences were checked using a sequence alignment editor (BioEdit v. 7.2.5^58^) and aligned through MUSCLE algorithm in MEGA7^59^. The alignments of three gene fragments were joined to the combined dataset using an online fasta sequence toolbox FaBox v. 1.41^60^.

Phylogenetic analyses were based on a five-partition dataset (3 codons of *COI* + *16S rRNA* + *28S rRNA*) of 147 sequences. Four representatives of the subfamily Gonideinae were used as outgroup, i.e. *Gonidea angulata* (Lea, 1838), *Potomida littoralis* (Cuvier, 1798), *Leguminaia wheatleyi* (Lea, 1862), and *Lamprotula leaii* (Gray in Griffith & Pidgeon, 1833) (Supplementary Table 1). The COI sequence of *B. exilis* (ICH-00734) was deleted from the dataset due to having some doubtful regions. The maximum likelihood (ML) analysis was performed through IQ-TREE (W-IQ-TREE) server^61^ using an automatic identification of most appropriate evolutionary models^62^ and an ultrafast bootstrap (UFBoot) algorithm with 5000 replicates^63^. Models of sequence evolution for each partition calculated through Model Finder^64^ based on Bayesian Information Criterion (BIC) were following: 1st codon COI − F81 + I; 2nd codon COI − TPM2u + G + I; 3rd codon COI − TN + G + I; 28S − HKY + G + I; TIM2e + G. Bayesian inference analysis (BI) was carried out in MrBayes v. 3.2.7^65^ with four runs, each with three heated (temperature = 0.1) and one cold Markov chain, during 25 million generations and sampling every 1000th generation. The first 15% of trees were discarded as burn-in. The calculation was performed at San Diego Supercomputer Center through the CIPRES Science Gateway^66^. A trace analysis tool (Tracer v. 1.7) was used to check a convergence of the MCMC chains to a stationary distribution^67^. The effective sample size (ESS) for all the parameters was recorded as > 3000. To study the phylogeography of *Bineurus* and *Thaiconcha* species, a median joining network was constructed through Network v. 4.6.1.3 software^21^ based on 102 *COI* haplotypes (Supplementary Table 1). The Mekong basin was divided into three sections (upper, middle, and lower) based on fish migration scheme developed by Halls and Kshatriya^22, 68^. MEGA7^59^ was used to calculate uncorrected p-distances between species and to check the fixed nucleotide differences.

### Nomenclatural acts

The electronic edition of this article conforms to the requirements of the amended International Code of Zoological Nomenclature (ICZN), and hence the new names contained herein are available under that Code from the electronic edition of this article. This published work and the nomenclatural acts it contains have been registered in ZooBank (http://zoobank.org), the online registration system for the ICZN. The LSID for this publication is: http://zoobank.org/urn:lsid:zoobank.org:pub:EE6080B0-3444-46D8-B24B-4B5D6B97EF4C. The electronic edition of this paper was published in a journal with an ISSN, and has been archived and is available from PubMed Central.

## Supplementary Information

Supplementary Information.

## Data Availability

Freshwater mussel sampling for this study was approved under permission of the Department of Fisheries, National Inland Fisheries Institute, Bangkok, Thailand. Our samples from Thailand were taken under the export permission No. 11501110316100766 dated on 15 March 2018 issued by the Suvarnabhumi Airport Fish Inspection Office. The type series of the new species are available in the UF—Florida Museum of Natural History, Gainesville, USA, and RMBH—Russian Museum of Biodiversity Hotspots, Federal Center for Integrated Arctic Research, Russian Academy of Sciences, Arkhangelsk, Russia. The sequences generated in this study are available from GenBank. The accession number and collecting locality for each specimen are presented in Supplementary Table 1. Shell measurements and reference DNA sequences for the type series of new freshwater mussel species from Mekong basin are given in Table 3.

**Table 3:** Shell measurements and reference DNA sequences for the type series of new *Bineurus* and *Thaiconcha* species from Mekong basin.

Species	Status of specimen	Specimen voucher	Shell length, mm	Shell height, mm	Shell width, mm	NCBI’s GenBank acc. nos		
						*COI*	*16S rRNA*	*28S rRNA*
*B. loeiensis* sp. nov	Holotype	biv119_1	76.6	38.7	20.8	KX865879	KX865650	KX865750
*B. loeiensis* sp. nov	Paratype	biv119_2	60.2	31.4	17.1	KX865880	KX865651	KX865751
*B. loeiensis* sp. nov	Paratype	biv119_3	60.1	29.9	17.2	KX865881	KX865652	KX865752
*B. loeiensis* sp. nov	Paratype	biv119_4	36.8	19.5	9.3	KX865882	KX865653	KX865753
*B. loeiensis* sp. nov	Paratype	biv119_5	73.2	35.7	20.1	n/a	n/a	n/a
*B. loeiensis* sp. nov	Paratype	biv119_6	77.1	39.8	21.3	n/a	n/a	n/a
*B. loeiensis* sp. nov	Paratype	biv119_7	54.6	29.0	15.6	n/a	n/a	n/a
*B. loeiensis* sp. nov	Paratype	biv119_8	56.8	30.2	15.6	n/a	n/a	n/a
*B. loeiensis* sp. nov	Paratype	biv119_9	68.5	36.0	18.7	n/a	n/a	n/a
*B. loeiensis* sp. nov	Paratype	biv119_10	66.6	33.8	18.6	n/a	n/a	n/a
*B. loeiensis* sp. nov	Paratype	biv119_11	71.8	38.0	18.3	n/a	n/a	n/a
*T. munelliptica* sp. nov	Holotype	biv468_1	98.3	54.7	32.7	MN275064	MN307253	MN307194
*T. munelliptica* sp. nov	Paratype	biv468_2	95.1	53.8	33.5	MN275065	MN307254	MN307195
*T. munelliptica* sp. nov	Paratype	biv468_3	68.0	41.2	21.9	MN275066	MN307255	MN307196
*T. munelliptica* sp. nov	Paratype	biv462	62.7	39.4	19.8	MN275063	MN307252	MN307193
*T. munelliptica* sp. nov	Paratype	biv468_4	71.9	43.3	23.6	n/a	n/a	n/a
*T. munelliptica* sp. nov	Paratype	biv468_5	57.8	34.7	17.6	n/a	n/a	n/a
*T. munelliptica* sp. nov	Paratype	biv468_6	51.8	30.6	15.6	n/a	n/a	n/a
*T. munelliptica* sp. nov	Paratype	UF 507607 (2014-0580)	75.9	45.6	24.6	MW603621	n/a	n/a
*T. munelliptica* sp. nov	Paratype	UF 507607 (2014-0581)	80.9	43.7	26.0	MW603622	n/a	n/a
*T. munelliptica* sp. nov	Paratype	UF 507466 (ICH-02116)	77.7	47.3	24.9	MW603691	n/a	n/a
*T. munelliptica* sp. nov	Paratype	UF 507470 (ICH-02121)	94.4	56.9	35.1	MW603692	n/a	n/a
*T. thaiensis* sp. nov	Holotype	UF 567706 (2014-0700)	69.7	38.8	21.3	MW603630	n/a	MW647150
*T. thaiensis* sp. nov	Paratype	UF 507660 (2014-0701)	42.1	25.2	11.8	MW603631	n/a	n/a
*T. thaiensis* sp. nov	Paratype	UF 507660 (2014-0702)	78.6	46.3	27.0	MW603632	n/a	n/a
*T. thaiensis* sp. nov	Paratype	UF 507660 (2014-0703)	93.8	53.9	28.9	MW603633	n/a	n/a
*T. thaiensis* sp. nov	Paratype	UF 507660	74.2	40.9	24.0	n/a	n/a	n/a
*T. thaiensis* sp. nov	Paratype	UF 507660	71.1	40.4	23.6	n/a	n/a	n/a
*T. thaiensis* sp. nov	Paratype	UF 507660	67.6	41.4	22.1	n/a	n/a	n/a
*T. thaiensis* sp. nov	Paratype	UF 507660	70.6	42.8	23.1	n/a	n/a	n/a
*T. thaiensis* sp. nov	Paratype	UF 507660	73.1	42.1	24.3	n/a	n/a	n/a
*T. thaiensis* sp. nov	Paratype	UF 507660	73.6	42.3	25.6	n/a	n/a	n/a
*T. thaiensis* sp. nov	Paratype	UF 507660	50.1	29.2	14.7	n/a	n/a	n/a
*T. thaiensis* sp. nov	Paratype	UF 507660	44.2	25.9	12.8	n/a	n/a	n/a
*T. thaiensis* sp. nov	Paratype	UF 507660	44.1	25.7	12.7	n/a	n/a	n/a
*T. thaiensis* sp. nov	Paratype	UF 507660	41.4	24.5	11.0	n/a	n/a	n/a
*T. thaiensis* sp. nov	Paratype	UF 507660	39.3	24.0	10.8	n/a	n/a	n/a
*T. thaiensis* sp. nov	Paratype	UF 507660	49.0	28.2	14.2	n/a	n/a	n/a
*T. thaiensis* sp. nov	Paratype	UF 507660	34.0	20.5	9.1	n/a	n/a	n/a Shell measurements and reference DNA sequences for the type series of new *Bineurus* and *Thaiconcha* species from Mekong basin.
