# Supplementary material for: New freshwater mussels from two Southeast Asian genera Bineurus and Thaiconcha (Pseudodontini, Gonideinae, Unionidae)
Source: Sci Rep. 2021 May 10;11:8244. doi: 10.1038/s41598-021-87633-w (PMC8110783; doi:10.1038/s41598-021-87633-w)
Supplement: Supplementary file 1 — Supplementary Information. [file 41598_2021_87633_MOESM1_ESM.docx]

SUPPLEMENTARY INFORMATION

**New freshwater mussels from two Southeast Asian genera Bineurus and Thaiconcha (Pseudodontini, Gonideinae, Unionidae)**

Ekaterina S. Konopleva, Ivan N. Bolotov, John M. Pfeiffer, Ilya V. Vikhrev, Alexander V. Kondakov, Mikhail Yu. Gofarov, Alena A. Tomilova, Kitti Tanmuangpak & Sakboworn Tumpeesuwan

*Corresponding author: es.konopleva@gmail.com

**Contents**

**Supplementary Tables**

**Supplementary Table 1.** List of sequences used in this study

**Supplementary Figures**

**Supplementary Figure 1.** Bayesian phylogeny of the Pseudodontini based on the complete data set of mitochondrial and nuclear sequences (five partitions: three codons of COI + 16S rRNA + 28S rRNA)

**Supplementary Table 1.** List of sequences used in this study

| **Species** | **Locality** | **Specimen Voucher** | **NCBI’s GenBank acc. nos.** | | |
| --- | --- | --- | --- | --- | --- |
|  |  |  | ***COI*** | ***16S rRNA*** | ***28S rRNA*** |
| **UNIONIDAE Rafinesque, 1820** |  |  |  |  |  |
| **Gonideinae Ortmann, 1916** |  |  |  |  |  |
| **Pseudodontini Frierson, 1927** |  |  |  |  |  |
| ***Pseudodon* Gould, 1844** |  |  |  |  |  |
| *Pseudodon* cf. *inoscularis* (Gould, 1844) | Myanmar: Ayeyarwady River basin, a tributary of Lake Indawgyi | RMBH biv0110_5 | KX865858 | KX865859 | KX865860 |
| *Pseudodon* cf. *inoscularis* (Gould, 1844) | Myanmar: Ayeyarwady River basin, a tributary of Lake Indawgyi | RMBH biv0110_10 | KX865629 | KX865630 | KX865631 |
| *Pseudodon* cf. *inoscularis* (Gould, 1844) | Myanmar: Ayeyarwady River basin, a tributary of Lake Indawgyi | RMBH biv0110_11 | KX865730 | KX865731 | KX865732 |
| *Pseudodon bogani* Bolotov, Kondakov & Konopleva, 2017 | Myanmar: Sittaung River basin, Kanni River | RMBH biv0241_4 | MF352216 | MF352290 | MF352348 |
| *Pseudodon bogani* Bolotov, Kondakov & Konopleva, 2017 | Myanmar: Sittaung River basin, Kanni River | RMBH biv0241_5 | MF352217 | MF352291 | MF352349 |
| *Pseudodon bogani* Bolotov, Kondakov & Konopleva, 2017 | Myanmar: Sittaung River basin, Kanni River | RMBH biv0241_8 | MF352218 | MF352292 | MF352350 |
| *Pseudodon manueli* Konopleva, Kondakov & Vikhrev, 2017 | Myanmar: Sittaung River basin, Pyowne River | RMBH biv0246_1 | MF352228 | MF352300 | MF352358 |
| *Pseudodon manueli* Konopleva, Kondakov & Vikhrev, 2017 | Myanmar: Sittaung River basin, Pyowne River | RMBH biv0246_3 | MF352229 | MF352301 | MF352359 |
| *Pseudodon manueli* Konopleva, Kondakov & Vikhrev, 2017 | Myanmar: Sittaung River basin, Pyowne River | RMBH biv0246_8 | MF352230 | MF352302 | MF352360 |
| *Pseudodon salwenianus* (Gould, 1844) | Myanmar: Salween River basin, unnamed stream | RMBH biv0639_1 | MN275037 | MN307237 | MN307178 |
| *Pseudodon salwenianus* (Gould, 1844) | Myanmar: Salween River basin, Hlaingbwe Stream | RMBH biv0674_2 | MN275041 | MN307239 | MN307180 |
| *Pseudodon kayinensis* Bolotov et al., 2020 | Myanmar: Ataran River basin, Winyaw River | RMBH biv0618_3 | MN275045 | MN307240 | MN307181 |
| *Pseudodon kayinensis* Bolotov et al., 2020 | Myanmar: Ataran River basin, Zami River, Ko Du Kwe Stream | RMBH biv0637_1 | MN275046 | MN307241 | MN307182 |
| *Pseudodon kayinensis* Bolotov et al., 2020 | Myanmar: Hlaingbwe River basin, unnamed stream | RMBH biv0638_1 | MN275049 | MN307242 | MN307183 |
| ***Pilsbryoconcha* Simpson, 1900** |  |  |  |  |  |
| *Pilsbryoconcha compressa* (Martens, 1860) | Thailand: Mekong River basin, Huai Nam Khu Reservoir | RMBH biv0118 | KX865875 | KX865646 | KX865746 |
| *P. compressa* (Martens, 1860) | Thailand: Mekong River basin, artificial pond near the Ban Nong-Bua village | RMBH biv0116_2 | KX865873 | KX865644 | KX865745 |
| *P. compressa* (Martens, 1860) | Thailand: Mekong River basin, Chae stream | RMBH biv0478_1 | MW603695 | MW590674 | MW647152 |
| *P. compressa* (Martens, 1860) | Thailand: Mekong River basin, Chae stream | RMBH biv0478_2 | MW603696 | MW590675 | MW647153 |
| *P. lemeslei* (Morelet, 1875) | Vietnam | n/a | KX822657 | n/a | n/a |
| *P. exilis* (Lea, 1838) | Malaysia | X213 | KX051289 | n/a | n/a |
| ***Bineurus*** Simpson, 1900 |  |  |  |  |  |
| *Bineurus* *mouhotii* (Lea, 1863) | Laos: Mekong River basin, Nam Long River | biv182_2 | KX865876 | KX865647 | KX865747 |
| *B.* *mouhotii* (Lea, 1863) | Laos: Mekong River basin, Nam Pe River | biv183_4 | KX865877 | KX865648 | KX865748 |
| *B.* *mouhotii* (Lea, 1863) | Laos: Mekong River basin, Nam Long River | biv182_19 | KX865878 | KX865649 | KX865749 |
| *B.* *mouhotii* (Lea, 1863) | Laos: Mekong River basin, a tributary of Nam Fa River near Vieng Phou Kha | biv201_2 | KY561623 | KY561641 | KY561655 |
| *B.* *mouhotii* (Lea, 1863) | Laos: Mekong River basin, a tributary of Nam Fa River near Vieng Phou Kha | biv201_3 | KY561624 | KY561642 | KY561656 |
| *B.* *mouhotii* (Lea, 1863) | Laos: Mekong River basin, a tributary of Nam Fa River near Vieng Phou Kha | biv201_5 | KY561625 | KY561643 | KY561657 |
| *B.* *mouhotii* (Lea, 1863) | Laos: Mekong River basin, a tributary of Nam Fa River near Vieng Phou Kha | biv202_1 | KY561626 | KY561644 | KY561658 |
| *B.* *mouhotii* (Lea, 1863) | Thailand: Mekong River basin, at Rt. 211 bridge approx. 3 km south of Ban Muang | UF_507756 (ICH-00735) | MW603670 | n/a | n/a |
| *B.* *mouhotii* (Lea, 1863) | Thailand: Mekong River basin, at Rt. 211 bridge approx. 3 km south of Ban Muang | UF_507756 (ICH-00733) | MW603668 | n/a | n/a |
| *B. loeiensis* **sp. nov.** | Thailand: Mekong River basin, Loei River | biv119_1 | KX865879 | KX865650 | KX865750 |
| *B. loeiensis* **sp. nov.** | Thailand: Mekong River basin, Loei River | biv119_2 | KX865880 | KX865651 | KX865751 |
| *B. loeiensis* **sp. nov.** | Thailand: Mekong River basin, Loei River | biv119_3 | KX865881 | KX865652 | KX865752 |
| *B. loeiensis* **sp. nov.** | Thailand: Mekong River basin, Loei River | biv119_4 | KX865882 | KX865653 | KX865753 |
| *B. anodontinum* (Rochebrune, 1882) | Cambodia: Mekong River basin, Tonle Sekong River | UF 507391 (ICH-00445) | MW603639 | n/a | n/a |
| *B. anodontinum* (Rochebrune, 1882) | Cambodia: Mekong River basin, Tonle Sekong River | UF 507391 (ICH-00447) | MW603641 | n/a | n/a |
| *B. anodontinum* (Rochebrune, 1882) | Cambodia: Mekong River basin, Tonle Sekong River | UF 507391 (ICH-00450) | MW603644 | n/a | n/a |
| *B. anodontinum* (Rochebrune, 1882) | Cambodia: Mekong River basin, Tonle Sekong River | UF 507391 (ICH-00446) | MW603640 | n/a | n/a |
| *B. anodontinum* (Rochebrune, 1882) | Cambodia: Mekong River basin, Tonle Sekong River | UF 507391 (ICH-00448) | MW603642 | n/a | n/a |
| *B. anodontinum* (Rochebrune, 1882) | Cambodia: Mekong River basin, Tonle Sekong River | UF 507391 (ICH-00449) | MW603643 | n/a | n/a |
| *B. anodontinum* (Rochebrune, 1882) | Cambodia: Mekong River basin, Tonle Kong River | UF 507896 (ICH-00465) | MW603645 | n/a | n/a |
| *B. anodontinum* (Rochebrune, 1882) | Cambodia: Mekong River basin, Tonle Kong River | UF 507896 (ICH-00467) | MW603647 | n/a | n/a |
| *B. anodontinum* (Rochebrune, 1882) | Cambodia: Mekong River basin, upstream from Sambour | UF 507419 (ICH-00553) | MW603660 | n/a | n/a |
| *B. anodontinum* (Rochebrune, 1882) | Cambodia: Mekong River basin, downstream of Sandan | UF 507424 (ICH-00564) | MW603661 | n/a | n/a |
| *B. anodontinum* (Rochebrune, 1882) | Cambodia: Mekong River basin, downstream of Sandan | UF 507424 (ICH-00565) | MW603662 | n/a | n/a |
| *B. anodontinum* (Rochebrune, 1882) | Cambodia: Mekong River basin, tributary just north of Phumi Prêk Preah on AH11 | UF 507408 (ICH-00523) | MW603649 | n/a | n/a |
| *B. anodontinum* (Rochebrune, 1882) | Cambodia: Mekong River basin, tributary just north of Phumi Prêk Preah on AH11 | UF 507408 (ICH-00525) | MW603651 | n/a | n/a |
| *B. anodontinum* (Rochebrune, 1882) | Cambodia: Mekong River basin, tributary just north of Phumi Prêk Preah on AH11 | UF 507408 (ICH-00526) | MW603652 | n/a | n/a |
| *B. anodontinum* (Rochebrune, 1882) | Cambodia: Mekong River basin, tributary just north of Phumi Prêk Preah on AH11 | UF 507408 (ICH-00527) | MW603653 | n/a | n/a |
| *B. anodontinum* (Rochebrune, 1882) | Cambodia: Mekong River basin, tributary just north of Phumi Prêk Preah on AH11 | UF 507408 (ICH-00528) | MW603654 | n/a | n/a |
| *B. anodontinum* (Rochebrune, 1882) | Cambodia: Mekong River basin, tributary just north of Phumi Prêk Krieng on AH11 | UF 559262 (ICH-00537) | MW603655 | n/a | n/a |
| *B. anodontinum* (Rochebrune, 1882) | Cambodia: Mekong River basin, tributary just north of Phumi Prêk Krieng on AH11 | UF 559262 (ICH-00541) | MW603659 | n/a | n/a |
| *B. exilis* (Morelet, 1866) | Thailand: Mekong River basin, Mun River, up-stream of upper reservoir | biv474_1 | MN275052 | MN307243 | MN307184 |
| *B. exilis* (Morelet, 1866) | Thailand: Mekong River basin, Mun River, up-stream of upper reservoir | biv474_3 | MN275053 | MN307244 | MN307185 |
| *B. exilis* (Morelet, 1866) | Laos: Mekong River basin | NCSM84903 | KX822663 | n/a | KX822619 |
| *B. exilis* (Morelet, 1866) | Laos: Mekong River basin | UMMZ:304649 | KP795026 | KP795051 | KP795009 |
| *B. exilis* (Morelet, 1866) | Cambodia: Mekong River basin, Tonle Kong River | UF 567737 (ICH-00466) | MW603646 | n/a | n/a |
| *B. exilis* (Morelet, 1866) | Cambodia: Mekong River basin, Tonle Kong River | UF 567737 (ICH-00468) | MW603648 | n/a | MW647151 |
| *B. exilis* (Morelet, 1866) | Cambodia: Mekong River basin, Tonle Srepok River | UF 507381 (ICH-00412) | MW603636 | n/a | n/a |
| *B. exilis* (Morelet, 1866) | Cambodia: Mekong River basin, Tonle Srepok River | UF 507381 (ICH-00422) | MW603637 | n/a | n/a |
| *B. exilis* (Morelet, 1866) | Cambodia: Mekong River basin, Tonle Srepok River | UF 507381 (ICH-00423) | MW603638 | n/a | n/a |
| *B. exilis* (Morelet, 1866) | Cambodia: Mekong River basin, tributary just north of Phumi Prêk Krieng on AH11 | UF 507413 (ICH-00538) | MW603656 | n/a | n/a |
| *B. exilis* (Morelet, 1866) | Cambodia: Mekong River basin, tributary just north of Phumi Prêk Krieng on AH11 | UF 507413 (ICH-00539) | MW603657 | n/a | n/a |
| *B. exilis* (Morelet, 1866) | Cambodia: Mekong River basin, tributary just north of Phumi Prêk Krieng on AH11 | UF 507413 (ICH-00540) | MW603658 | n/a | n/a |
| *B. exilis* (Morelet, 1866) | Cambodia: Mekong River basin, tributary just north of Phumi Prêk Preah on AH11 | UF 559380 (ICH-00524) | MW603650 | n/a | n/a |
| *B. exilis* (Morelet, 1866) | Thailand: Mekong River basin, Dom Noi River | UF 507854 (ICH-01181) | MW603684 | n/a | n/a |
| *B. exilis* (Morelet, 1866) | Thailand: Mekong River basin, Dom Noi River | UF 507854 (ICH-01182) | MW603685 | n/a | n/a |
| *B. exilis* (Morelet, 1866) | Thailand: Mekong River basin, Dom Noi River | UF 507854 (ICH-01183) | MW603686 | n/a | n/a |
| *B. exilis* (Morelet, 1866) | Thailand: Mekong River basin, Dom Noi River | UF 507854 (ICH-01185) | MW603687 | n/a | n/a |
| *B. exilis* (Morelet, 1866) | Thailand: Mekong River basin, Dom Noi River | UF 507854 (ICH-01186) | MW603688 | n/a | n/a |
| *B. exilis* (Morelet, 1866) | Thailand: Mekong River basin, Songkhram River at confluence with Mao River | UF 507639 (2014-0662) | MW603625 | n/a | n/a |
| *B. exilis* (Morelet, 1866) | Thailand: Mekong River basin, Songkhram River at confluence with Mao River | UF 507639 (2014-0663) | MW603626 | n/a | n/a |
| *B. exilis* (Morelet, 1866) | Thailand: Mekong River basin, Songkhram River at confluence with Yam River | UF 507644 (2014-0668) | MW603627 | n/a | n/a |
| *B. exilis* (Morelet, 1866) | Thailand: Mekong River basin, Songkhram River at confluence with Yam River | UF 507644 (2014-0669) | MW603628 | n/a | n/a |
| *B. exilis* (Morelet, 1866) | Thailand: Mekong River basin, Songkhram River at confluence with Yam River | UF 507644 (2014-0670) | MW603629 | n/a | n/a |
| *B. exilis* (Morelet, 1866) | Cambodia: Mekong River basin, Tributary of Tonle Sap River | UF 507440 (ICH-00609) | MW603663 | n/a | n/a |
| *B. exilis* (Morelet, 1866) | Cambodia: Mekong River basin, Tributary of Tonle Sap River | UF 507440 (ICH-00610) | MW603664 | n/a | n/a |
| *B. exilis* (Morelet, 1866) | Cambodia: Mekong River basin, Tributary of Tonle Sap River | UF 507440 (ICH-00611) | MW603665 | n/a | n/a |
| *B. exilis* (Morelet, 1866) | Thailand: Mekong River basin, Bang Sai River | UF 507816 (ICH-00856) | MW603671 | n/a | n/a |
| *B. exilis* (Morelet, 1866) | Thailand: Mekong River basin, Bang Sai River | UF 507816 (ICH-00857) | MW603672 | n/a | n/a |
| *B. exilis* (Morelet, 1866) | Thailand: Mekong River basin, Bang Sai River | UF 507816 (ICH-00858) | MW603673 | n/a | n/a |
| *B. exilis* (Morelet, 1866) | Thailand: Mekong River basin, Bang Sai River | UF 507816 (ICH-00859) | MW603674 | n/a | n/a |
| *B. exilis* (Morelet, 1866) | Thailand: Mekong River basin, Bang River | UF 507828 (ICH-00881) | MW603675 | n/a | n/a |
| *B. exilis* (Morelet, 1866) | Thailand: Mekong River basin, Bang River | UF 507828 (ICH-00882) | MW603676 | n/a | n/a |
| *B. exilis* (Morelet, 1866) | Thailand: Mekong River basin, Bang River | UF 507828 (ICH-00883) | MW603677 | n/a | n/a |
| *B. exilis* (Morelet, 1866) | Thailand: Mekong River basin, Bang River | UF 507835 (ICH-00894) | MW603678 | n/a | n/a |
| *B. exilis* (Morelet, 1866) | Thailand: Mekong River basin, Bang River | UF 507835 (ICH-00895) | MW603679 | n/a | n/a |
| *B. exilis* (Morelet, 1866) | Thailand: Mekong River basin, Sa Thung River | UF 507845 (ICH-01158) | MW603680 | n/a | n/a |
| *B. exilis* (Morelet, 1866) | Thailand: Mekong River basin, Ta Wang River | UF 507846 (ICH-01159) | MW603681 | n/a | n/a |
| *B. exilis* (Morelet, 1866) | Thailand: Mekong River basin, Ta Wang River | UF 507846 (ICH-01160) | MW603682 | n/a | n/a |
| *B. exilis* (Morelet, 1866) | Thailand: Mekong River basin, Ta Wang River | UF 507846 (ICH-01161) | MW603683 | n/a | n/a |
| *B. exilis* (Morelet, 1866) | Thailand: Mekong River basin, Plai Mat River | UF 507476 (ICH-02133) | MW603693 | n/a | n/a |
| *B. exilis* (Morelet, 1866) | Thailand: Mekong River basin, Plai Mat River | UF 507476 (ICH-02134) | MW603694 | n/a | n/a |
| *B. exilis* (Morelet, 1866) | Thailand: Mekong River basin, at Rt. 211 bridge approx. 3 km south of Ban Muang | UF 541633 (ICH-00734) | MW603669 | n/a | n/a |
| ***Thaiconcha* Bolotov et al., 2020** |  |  |  |  |  |
| *Thaiconcha callifera* (Martens, 1860) | Thailand: Mekong River basin, Phong River | biv_120_4 | KX865862 | KX865633 | KX865734 |
| *T. callifera* (Martens, 1860) | Thailand: Mekong River basin, Phong River | biv_120_12 | KX865863 | KX865634 | KX865735 |
| *T. callifera* (Martens, 1860) | Thailand: Mekong River basin, Phong River | biv_120_15 | KX865864 | KX865635 | KX865736 |
| *T. callifera* (Martens, 1860) | Thailand: Mekong River basin, Phong River | biv_120_3 | KX865865 | KX865636 | KX865737 |
| *T. callifera* (Martens, 1860) | Thailand: Mekong River basin, Phong River | biv_120_8 | KX865866 | KX865637 | KX865738 |
| *T. callifera* (Martens, 1860) | Thailand: Mekong River basin, Phong River | biv_120_14 | KX865867 | KX865638 | KX865739 |
| *T. callifera* (Martens, 1860) | Thailand: Mekong River basin, Phong River | biv_120_7 | KX865868 | KX865639 | KX865740 |
| *T. callifera* (Martens, 1860) | Thailand: Mekong River basin, Phong River | biv_120_11 | KX865869 | KX865640 | KX865741 |
| *T. callifera* (Martens, 1860) | Thailand: Mekong River basin, Phong River | biv_120_13 | KX865870 | KX865641 | KX865742 |
| *T. callifera* (Martens, 1860) | Thailand: Mekong River basin, Phong River | biv_205_4 | KY561622 | KY561640 | KY561654 |
| *T. callifera* (Martens, 1860) | Thailand: Mekong River basin, Kong Si River | UF 507741 (ICH-00712) | MW603666 | n/a | n/a |
| *T. callifera* (Martens, 1860) | Thailand: Mekong River basin, Kong Si River | UF 507741 (ICH-00714) | MW603667 | n/a | n/a |
| *T. callifera* (Martens, 1860) | Thailand: Mekong River basin, Dom Yai River | UF 507860 (ICH-01192) | MW603689 | n/a | n/a |
| *T. callifera* (Martens, 1860) | Thailand: Mekong River basin, Dom Yai River | UF 507860 (ICH-01193) | MW603690 | n/a | n/a |
| *T. callifera* (Martens, 1860) | Thailand: Mekong basin, Mun drainage, Pao River | UF 507621 (2014-0617) | MW603623 | n/a | n/a |
| *T. callifera* (Martens, 1860) | Thailand: Mekong basin, Mun drainage, Pao River | UF 507621 (2014-0618) | MW603624 | n/a | n/a |
| *T. munelliptica* **sp. nov** | Thailand: Mekong River basin, Mun River | biv462 | MN275063 | MN307252 | MN307193 |
| *T. munelliptica* **sp. nov** | Thailand: Mekong River basin, Mun River | biv468_1 | MN275064 | MN307253 | MN307194 |
| *T. munelliptica* **sp. nov** | Thailand: Mekong River basin, Mun River | biv468_2 | MN275065 | MN307254 | MN307195 |
| *T. munelliptica* **sp. nov** | Thailand: Mekong River basin, Mun River | biv468_3 | MN275066 | MN307255 | MN307196 |
| *T. munelliptica* **sp. nov** | Thailand: Mekong River basin, Mun River | UF 507607 (2014-0580) | MW603621 | n/a | n/a |
| *T. munelliptica* **sp. nov** | Thailand: Mekong River basin, Mun River | UF 507607 (2014-0581) | MW603622 | n/a | n/a |
| *T. munelliptica* **sp. nov** | Thailand: Mekong River basin, Mun River | UF 507466 (ICH-02116) | MW603691 | n/a | n/a |
| *T. munelliptica* **sp. nov** | Thailand: Mekong River basin, Chi River | UF 507470 (ICH-02121) | MW603692 | n/a | n/a |
| *T. thaiensis* **sp. nov.** | Thailand: Mekong River basin, Kham Nong Bua River | UF 567706 (2014-0700) | MW603630 | n/a | MW647150 |
| *T. thaiensis* **sp. nov.** | Thailand: Mekong River basin, Kham Nong Bua River | UF 507660 (2014-0701) | MW603631 | n/a | n/a |
| *T. thaiensis* **sp. nov.** | Thailand: Mekong River basin, Kham Nong Bua River | UF 507660 (2014-0702) | MW603632 | n/a | n/a |
| *T. thaiensis* **sp. nov.** | Thailand: Mekong River basin, Kham Nong Bua River | UF 507660 (2014-0703) | MW603633 | n/a | n/a |
| *T. thaiensis* **sp. nov.** | Thailand: Chao Phraya River basin, Wang River | UF 507663 (2014-0708) | MW603634 | n/a | n/a |
| *T. thaiensis* **sp. nov.** | Thailand: Chao Phraya River basin, Wang River | UF 507663 (2014-0709) | MW603635 | n/a | n/a |
| ***Monodontina* Conrad, 1853** |  |  |  |  |  |
| *Monodontina vondembuschiana (*Lea, 1840) | Malaysia | BIV1721 | KX051296 | n/a | n/a |
| *M. vondembuschiana (*Lea, 1840) | Malaysia | BIV1806 | KX051303 | n/a | n/a |
| *M. vondembuschiana (*Lea, 1840) | Malaysia | X157 | KX051306 | n/a | n/a |
| *M. mekongii* Bolotov et al., 2020 | Thailand: Mekong River basin, Phong River | RMBH biv0122 | KX865861 | KX865632 | KX865733 |
| *M. laosica* Bolotov et al., 2020 | Laos: Mekong River basin, tributary of the Vang Ngao River | UMMZ 304650 | KP795029 | KP795052 | n/a |
| *M. cambodiensis* (Petit de la Saussaye, 1865) | Cambodia: Mekong River basin, Tonle Sap River: Pursat River | UMMZ 304350 | KP795028 | KF011262 | KP795011 |
| *M. cambodiensis* (Petit de la Saussaye, 1865) | Malaysia | X198 | KX051297 | n/a | n/a |
| *M. cambodiensis* (Petit de la Saussaye, 1865) | Thailand | n/a | KX822660 | n/a | KX822616 |
| *M. lenyanensis* Bolotov et al., 2020 | Myanmar: Lenya River basin, 14 Mile Stream | RMBH biv0628_1 | MN275054 | MN307245 | MN307186 |
| *M. lenyanensis* Bolotov et al., 2020 | Myanmar: Lenya River basin, 14 Mile Stream | RMBH biv0628_2 | MN275055 | MN307246 | MN307187 |
| *M. lenyanensis* Bolotov et al., 2020 | Myanmar: Lenya River basin, 14 Mile Stream | RMBH biv0628_3 | MN275056 | MN307247 | MN307188 |
| ***Sundadontina* Bolotov et al., 2020** |  |  |  |  |  |
| *Sundadontina cumingii* (Lea, 1850) | Malaysia | X115 | KX051295 | n/a | n/a |
| *S. cumingii* (Lea, 1850) | Malaysia | X79 | KX051292 | n/a | n/a |
| *S. tumida* (Morelet, 1866) | Cambodia: Mekong River basin | UMMZ 304349 | KP795027 | KF011261 | KP795010 |
| *S. tanintharyiensis* Bolotov et al., 2020 | Myanmar: Lenya River basin, Chaung Nauk Pyan Stream | RMBH biv0643_4 | MN275057 | MN307248 | MN307189 |
| *S. brandti* Bolotov et al., 2020 | Thailand: Mekong River basin, Mun River | RMBH biv0475_2 | MN275058 | MN307249 | MN307190 |
| *S. brandti* Bolotov et al., 2020 | Thailand: Mekong River basin, Mun River | RMBH biv0475_3 | MN275059 | MN307250 | MN307191 |
| *S. brandti* Bolotov et al., 2020 | Thailand: Mekong River basin, Mun River | RMBH biv0475_4 | MN275060 | n/a | n/a |
| *S. taskaevi* Bolotov et al., 2020 | Thailand: Mekong River basin, Mun River | RMBH biv0475_1 | MN275061 | MN307251 | MN307192 |
| *S. taskaevi* Bolotov et al., 2020 | Thailand: Mekong River basin, Mun River | RMBH biv0475_5 | MN275062 | n/a | n/a |
| ***Nyeinchanconcha* Bolotov et al., 2020** |  |  |  |  |  |
| *Nyeinchanconcha nyeinchani* Bolotov et al., 2020 | Laos: Mekong River basin, Nam Phiat River | UMMZ 304648 | KP795025 | KP795050 | KP795008 |
| *N. nyeinchani* Bolotov et al., 2020 | Laos: Mekong River basin | NCSM 84884 | KX822662 | n/a | KX822618 |
| **Out-group taxa** |  |  |  |  |  |
| *Gonidea angulata* (Lea, 1838) | USA: Okanagan Lake | RMBH biv0294_1 | MN402615 | MN396726 | MN396722 |
| *Potomida littoralis* (Cuvier, 1798) | Turkey: Karasu River | RMBH biv0177_10 | MN402617 | MN396728 | MN396724 |
| *Leguminaia wheatleyi* (Lea, 1862) | Turkey: Karasu River | RMBH biv0177_7 | MN402614 | MN396725 | MN396721 |
| *Lamprotula leaii* (Griffith & Pidgeon, 1833) | Vietnam | RMBH biv0200_1 | MN402616 | MN396727 | MN396723 |

n/a – not available.


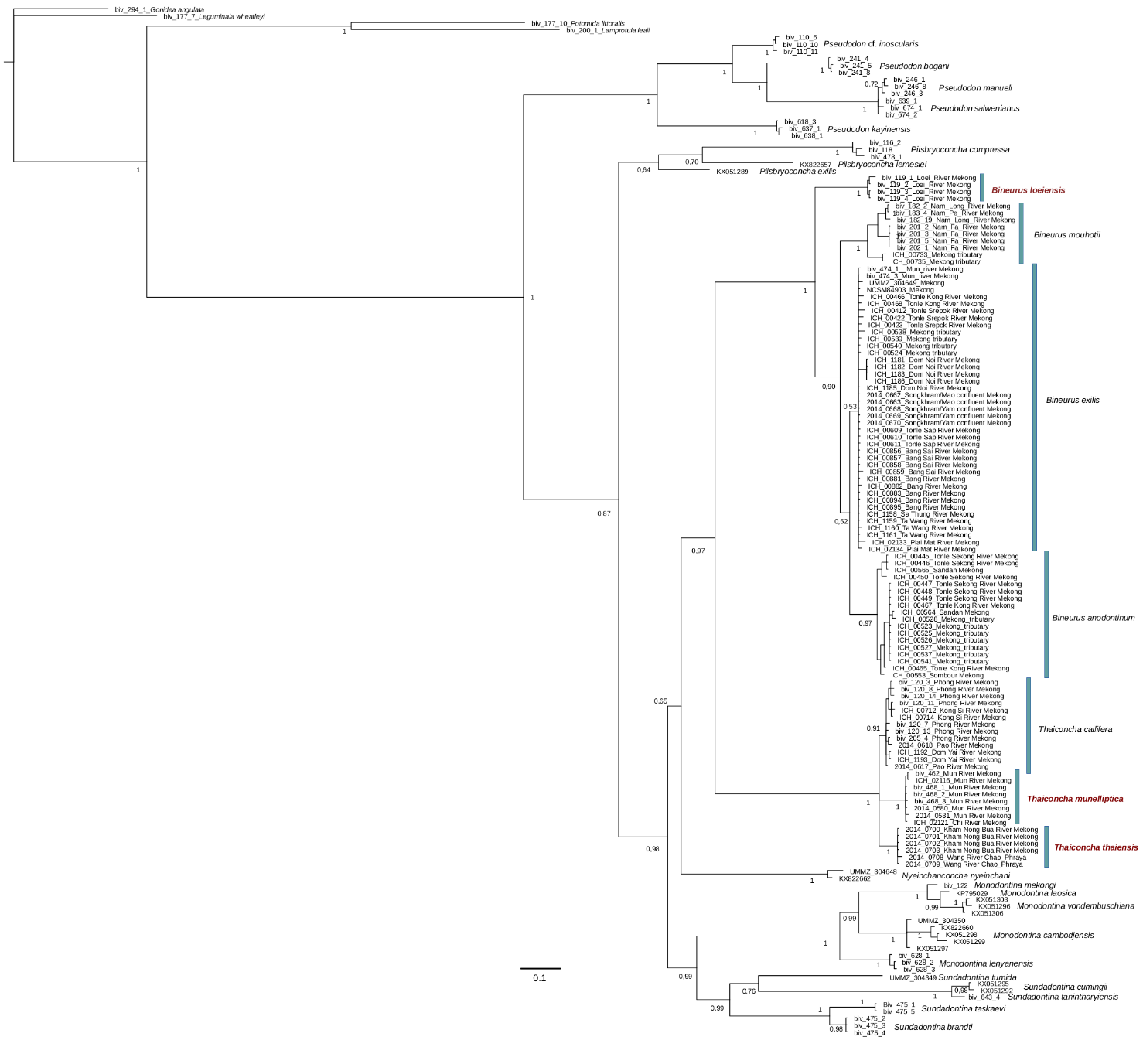


**Supplementary Figure 1.** Bayesian phylogeny of the Pseudodontini based on the complete data set of mitochondrial and nuclear sequences (five partitions: three codons of COI + 16S rRNA + 28S rRNA)
